# Identification of STAT1 and STAT3 Specific Inhibitors Using Comparative Virtual Screening and Docking Validation

**DOI:** 10.1371/journal.pone.0116688

**Published:** 2015-02-24

**Authors:** Malgorzata Szelag, Anna Czerwoniec, Joanna Wesoly, Hans A. R. Bluyssen

**Affiliations:** 1 Department of Human Molecular Genetics, Institute of Molecular Biology and Biotechnology, Adam Mickiewicz University in Poznan, Umultowska 89, 61-614 Poznan, Poland; 2 Laboratory of High Throughput Technologies, Institute of Molecular Biology and Biotechnology, Adam Mickiewicz University in Poznan, Umultowska 89, 61-614 Poznan, Poland; 3 Bioinformatics Laboratory, Institute of Molecular Biology and Biotechnology, Adam Mickiewicz University in Poznan, Umultowska 89, 61-614 Poznan, Poland; University of Regensburg, GERMANY

## Abstract

Signal transducers and activators of transcription (STATs) facilitate action of cytokines, growth factors and pathogens. STAT activation is mediated by a highly conserved SH2 domain, which interacts with phosphotyrosine motifs for specific STAT-receptor contacts and STAT dimerization. The active dimers induce gene transcription in the nucleus by binding to a specific DNA-response element in the promoter of target genes. Abnormal activation of STAT signaling pathways is implicated in many human diseases, like cancer, inflammation and auto-immunity. Searches for STAT-targeting compounds, exploring the phosphotyrosine (pTyr)-SH2 interaction site, yielded many small molecules for STAT3 but sparsely for other STATs. However, many of these inhibitors seem not STAT3-specific, thereby questioning the present modeling and selection strategies of SH2 domain-based STAT inhibitors. We generated new 3D structure models for all human (h)STATs and developed a comparative *in silico* docking strategy to obtain further insight into STAT-SH2 cross-binding specificity of a selection of previously identified STAT3 inhibitors. Indeed, by primarily targeting the highly conserved pTyr-SH2 binding pocket the majority of these compounds exhibited similar binding affinity and tendency scores for all STATs. By comparative screening of a natural product library we provided initial proof for the possibility to identify STAT1 as well as STAT3-specific inhibitors, introducing the ‘STAT-comparative binding affinity value’ and ‘ligand binding pose variation’ as selection criteria. *In silico* screening of a multi-million clean leads (CL) compound library for binding of all STATs, likewise identified potential specific inhibitors for STAT1 and STAT3 after docking validation. Based on comparative virtual screening and docking validation, we developed a novel STAT inhibitor screening tool that allows identification of specific STAT1 and STAT3 inhibitory compounds. This could increase our understanding of the functional role of these STATs in different diseases and benefit the clinical need for more drugable STAT inhibitors with high specificity, potency and excellent bioavailability.

## Introduction

Cytokines and growth factors are the main tool of the organism to battle any kind of immune challenge like inflammation or cancer. Signal transducers and activators of transcription (STATs) are targets for activation by many of these signals, including interferons (IFNs), interleukins (ILs) and growth factors like EGF (Epidermal Growth Factor) and PDGF (Platelet-Derived Growth Factor). Also oncoproteins ABL (Abelson murine leukemia viral oncogene homolog) and Src are STAT activators. The STAT family is composed of seven members: STAT1, STAT2, STAT3, STAT4, STAT5A, STAT5B and STAT6. Structurally they share five domains, which are an amino-terminal domain, a coiled-coil domain, a DNA-binding domain, a SH2 (Src Homology 2) domain and a carboxyl-terminal transactivation domain [1]. STAT activation is mediated by a highly conserved SH2 domain, which interacts with phosphotyrosine (pTyr) motifs for specific STAT-receptor contacts and STAT dimerization. The active dimers induce gene transcription in the nucleus by binding to a specific DNA-response element in the promoter of target genes [2]. STAT proteins promote fundamental cellular processes, including cell growth and differentiation, development, apoptosis, immune responses and inflammation. STATs are convergence points of many oncogenic and inflammatory pathways, therefore, the abnormal activation of STAT signaling pathways is also implicated in many human diseases. Especially STAT1 and STAT3 display prominent roles in cancer, inflammation and auto-immunity. STAT1 is responsible for cell growth and apoptosis, T_H_1 cell-specific cytokine production and antimicrobial defense. It plays tumor-suppresive function and has pro-atherogenic properties. Atypical STAT1 activation leads to cardiovascular diseases like atherosclerosis, whereas STAT1 deficiency is responsible for causing infections and immune disorders. STAT3 function is essential for early embryonic development, cell proliferation and survival, inflammation and immune response, as well as cell motility. STAT3 function is often aberrant in the context of cancer. Constitutively active STAT3 is detected in numerous malignancies, including breast, melanoma, prostate, head and neck squamous cell carcinoma (HNSCC), multiple myeloma, pancreatic, ovarian, and brain tumours. There is growing evidence that preternatural functioning of other STATs also leads to immune disorders and infections (STAT2), autoimmune diseases like lupus (STAT4), chronic myelogenous leucaemia (STAT5A and STAT5B), as well as astma and allergy (STAT6). STAT inhibitors therefore could be valuable in treatment of these diseases [3–6].

Various STAT inhibitory strategies are being pursued, particularly for STAT3, including disruption of dimerization, tyrosine kinase STAT-competitive inhibitors, decoy deoxyrybonucleotides blocking STAT-DNA binding, induction of protein tyrosine phosphatases which dephosphorylate STATs and antisense oligonucleotides targeting STAT-mRNAs. Amid these approaches most studies focus on inhibiting STAT dimerization using small molecules identified by molecular modeling, virtual screening, computer-aided drug design, organometallic compounds or natural products [7–10]. According to the crystal structure of murine STAT3β, pTyr705, localized at the border of SH2 and transactivation domain, in one STAT3 monomer binds to the SH2 domain of the other [11]. Moreover, the SH2 domain comprises of several sub-pockets that can be targeted by small-molecule inhibitors, including: (1) pTyr705-binding pocket or pY+0, and (2) a hydrophobic side-pocket or pY-X [12]. Since dimerization via reciprocal phosphotyrosine-SH2 interactions is a key event in the activation of STATs, manipulations disrupting the dimer formation, such as use of small molecules, render the protein incapable of forming dimers, binding DNA and inducing gene transcription [13]. Disruption of e.g. STAT3 dimer formation therefore provides an effective therapeutic approach in cancer by blocking its aberrant signaling hyperactivity and pro-oncogenic effects [14].

Searches for STAT3-targeting compounds, exploring the pTyr-SH2 interaction area of STAT3, are numerous and yielded many small molecules. For example, STA-21 discovered by structure-based virtual screening was one of the first reported small inhibitors. It inhibits STAT3 dimerization, DNA binding, and STAT3-dependent transcription in breast cancer cells [15]. Another small molecule, stattic, was discovered by high-throughput screening and has been shown to selectively inhibit activation, dimerization, nuclear translocation of STAT3, and to increase apoptosis in STAT3-dependent cancer cell lines [16]. Among all the reported non-peptidomimetic small inhibitors, 5-hydroxy-9,10-dioxo-9,10-dihydroanthracene-1-sulfonamide (LLL12) has the lowest IC50 (0.16−3.09 μM), inhibiting STAT3 phosphorylation and the growth of human cancer cells [17]. Natural products have been an important resource in STAT3 inhibitor discovery and these efforts have yielded several lead candidates, including curcumin and resveratrol [18,19]. In many of these cases, however, the mechanism of action of these candidates with regard to STAT3 activity is unclear. It is possible that they inhibit STAT3 indirectly and are likely to block several targets [10].

However, it becomes clear that many of these inhibitors are not STAT3-specific, thereby questioning the present selection strategies of SH2 domain-based competitive small inhibitors for STAT3 and other STATs. The virtual screening approaches are mostly based on the limited available crystallographic data from STAT1 and STAT3 dimers. Therefore, no valid comparative information exists about differences and commonalities between STAT-SH2 domains and their detailed interactions with small compound inhibitors. Indeed, comparative information concerning pY+0 and pY-X in the SH2 domain of all STATs is lacking and cross-binding specificity of previously identified STAT3 inhibitors has not been properly checked. Together, this illustrates the need for better models and screening and docking validation tools that allow the identification of STAT-specific inhibitors.

Here, we generated 3D structure models for all human (h)STATs (1, 2, 3, 4, 5A, 5B and 6) and developed a STAT inhibitor screening method, based on comparative *in silico* virtual screening and docking validation, to obtain further insight into STAT-SH2 cross-binding specificity of a selection of previously identified STAT3 inhibitors. The standard selection criteria of these compounds were, confirmed *in vitro*, disruption of the phosphotyrosine-SH2 interactions and proven STAT cross-binding. By comparative virtual screening of a natural compound and clean leads library for binding of all STATs and introducing the ‘STAT-comparative binding affinity value’ (STAT-CBAV) and ‘ligand binding pose variation’ (LBPV) parameter as selection criteria, we provide initial proof that this novel *in silico* screening method enables selection of STAT3 as well as STAT1-specific inhibitors.

## Methods

### Sequence analysis

First, searches of the reference version of current sequence database (refseq_protein) for vertebrates were carried out at the NCBI using BLASTp [20] with default parameters and e-value threshold of 1e-3. Full-length sequences of *H*. *sapiens* STAT1 isoform alpha, STAT2 isoform 1, STAT3 isoform 1, STAT4, STAT5A, STAT5B and STAT6 isoform 1 (NCBI gene identification numbers 6274552, 4885615, 21618340, 4507255, 21618342, 21618344 and 23397678, respectively) were used as queries. The multiple sequence alignment (MSA) of human (h)STATs and homologous proteins identified in the database was calculated using MUSCLE [21], with default parameters, and refined manually to ensure that no unwarranted gaps had been introduced within α-helices and β-strands. On the basis of the alignment, phylogenetic tree was calculated with MEGA 6 [22] employing the Neighbor Joining method with JTT model of substitutions and pairwise deletions. The stability of individual nodes was calculated by the bootstrap test (1000 replicates).

### Local structure prediction and protein fold-recognition

Full-length sequences of hSTATs were submitted to Genesilico Metaserver gateway [23] to predict the protein structure by different fold-recognition methods (e.g. HHsearch [24] mGenTHREADER [25] and COMPASS [26]), which were compared, evaluated, and ranked by the Pcons5 [27]. This analysis revealed protein domain composition, sequence based features and templates for homology modeling. We generated new 3D structure models for all hSTATs (1, 2, 3, 4, 5A, 5B and 6) based on the published crystal structures of STAT1 (PDB Id`s: 1YVL, 1BF5), STAT3 (PDB Id: 1BG1), STAT4 (PDB Id: 1BGF) and STAT5A (PDB Id: 1Y1U), applying the homology modeling procedure.

In case of STAT1 protein we used comparative analysis to build a 3D structure of maximal length with a flexible linker and selected two templates for modeling—1YVL [28] (crystal structure of unphosphorylated STAT1 monomer) and 1BF5 [29] (crystal structure of a tyrosine phosphorylated STAT1 dimer). As suggested by these fold-recognition methods we used the same crystal structures for modeling of STAT2 protein. For STAT3 protein we took information from the crystal structure of the STAT3β homodimer bound to DNA [11] (PDB Id: 1BG1) and for missing N-terminal domain, 1YVL and 1BF5 crystal structures of STAT1. Similar procedure was applied for modeling of STAT4, where we exerted the crystal structure of the amino-terminal protein interaction domain of STAT4 [29] (PDB Id: 1BGF) and for missing regions the crystal structures of 1YVL and 1BF5 were used. To obtain full-length models of STAT5A, 5B and 6 we employed a crystal structure of the unphosphorylated STAT5A dimer [30] (PDB Id: 1Y1U) together with 1YVL.

### hSTAT protein homology modeling protocol

Preliminary models of the individual STAT monomers were built with MODELLER [31] based on the sequence alignment between hSTAT proteins and the template structures obtained from folds at the top positions of the Pcons5 ranking [27]. For all STATs models we also built pTyr-linkers, which play a crucial role in the dimerization process. For STAT1 and STAT3 this was based on available structural information. Structure of phosphotyrosine linkers for STAT1 (aa 700–710: G**pY^701^**IKTELISVS) and STAT3 (aa 704–714: P**pY^705^**LKTKFICVT) were obtained from the two available X-ray crystal models (PDB code: 1BF5 and 1BG1). The preliminary models of pTyr-linkers for STAT2 (aa 689–699: K**pY^690^**LKHRLIVVS), STAT4 (aa 692–702: G**pY^693^**VPSVFIPIS), STAT5A (aa 693–703: G**pY^694^**VKPQIKQVV), STAT5B (aa 698–708 G**pY^699^**VKPQIKQVV) and STAT6 (aa 640–650 G**pY^641^**VPATIKMTV) were built with MODELLER [31] based on the sequence alignment between STATs and the template structure 1BF5-linker of STAT1. Then, subsequently optimized at the semi-empirical PM3MM [32] level of theory, including the molecular mechanics correction for peptide linkage, using Gaussian 09 suite [33].

### hSTAT models refinement

All preliminary models were assessed with MetaMQAP [34] to predict their accuracy at the level of individual residues. Hybrid models were then refined in poorly scored regions (mainly loop regions) with REFINER (with restraints on remaining predicted secondary structure) [35] and SuperLooper [36] programs. After refinement, homology models of hSTATs combined with their linkers underwent a two-step energy minimization process in AMBER force field in HyperChem software. First, steepest descent algorithm to the RMS gradient value 1.0 kcal/(mol x Å) was used and secondly Polak-Ribiere conjugate gradient algorithm to the RMS gradient value 0.1 kcal/(mol x Å). Finally Chiron Server [37] was used to perform a rapid equilibration of all human STAT models using discrete molecular dynamics with an all-atom representation for each residue in the protein.

Final models were again evaluated with MetaMQAP and PROQ [38] methods to assess quality improvements. Thus, our structural predictions are highly accurate and can be used as receptors in docking simulations [39–42]. Models and their features were visualized with PyMOL [43]. Mapping of the electrostatic potential on protein surfaces was calculated with adaptive Poisson–Boltzmann solver (APBS) [44]. Moreover, employing results from MSA and phylogenetic relations the evolutionary conservation of amino acid positions in hSTATs was estimated with ConSurf server [45].

### Small inhibitor preparation

Small compounds used for docking—Cucurbitacin E and Cucurbitacin Q [46], Curcumin [47], LLL12 [17], Cpd188, Cpd30–12 [48], Stattic [16], STX-0119 [49], S3I-201 [50], S3I-201.1066 [51], BP-1–102 [19], WP1066 [52] and recently reported—FLLL32 [53], HJC0123 [54] and OPB-31121 [55] were built using GaussView 5.0 and optimized at the hybrid meta density functional theory (DFT) level—M05–2X/6–31G(d,p) [56] with tight convergence criteria and vibration frequency analysis. The calculations were carried out using Gaussian 09 suite [33]. The final optimized structures of the ligands were in their local energy minima, which were confirmed by absence of imaginary vibrational frequencies. The selection criteria of these compounds were: (1) bioavailability (e.g. WP1066), (2) discovery by molecular modeling or chemical modifications (e.g. FLLL32, HJC0123, S3I-201.1066), virtual or library screening (e.g. Cpd188, Cpd30–12, LLL12, STX-0119), or natural products (e.g. curcumin, cucurbitacin E and Q), (3) confirmed STAT3 phosphorylation inhibition *in vitro* and/or *in vivo* with low IC50 (e.g. FLLL32, LLL12), (4) possible oral administration (e.g. BP-1–102, OPB-31121, HJC0123), (5) possibility of STAT cross-binding (e.g. S3I-201, S3I-201.1066, curcumin, Cpd30–12).

### Compound libraries

Two small compound libraries were selected from ZINC Database (http://zinc.docking.org): (1) natural products (NP, 13 company catalogs with natural products—AfroDb Natural Products, AnalytiCon Discovery NP, Herbal Ingredients In-Vivo Metabolism, Herbal Ingredients Targets, IBScreen NP, Indofine Natural Products, NPACT Database, Nubbe Natural Products, Princeton NP, Selleck BioChemicals NP, Specs Natural Products, TCM Database @ Taiwan, UEFS Natural Products; subset size—131 582; criteria of selection—recognized by at least two proteins: the end of their biosynthetic pathway and their evolutionary biological target; update on 05.11.2013) and (2) clean leads (subset size—5 735 035 compounds; criteria of selection—molar weight [250;350], atomic based partition coefficient (xlogp) ≤ 3.5 and rotatable bonds ≤ 7; update on 31.01.2014).

### Comparative docking of STAT3 inhibitors

First, the AMBER ff99SB charges were applied to all models of human STAT proteins and STAT3 inhibitory compounds optimized by M05-2X/6-31G(d,p). In comparative docking procedure on the level of protein structures we selected the highly conserved pTyr binding pocket (pY+0) and hydrophobic side-pocket (pY-X) on the surface of SH2 domain. Then ligand-based approach was used to generate a ‘protomol’—molecular probe which is a 3D representation of the active site to which ligands are matched. In case of STAT proteins the ligand used to generate a ‘protomol’ was fragment of STAT-SH2 specific pTyr-linker matching to the selected sub-pockets: for STAT1 (G**pY^701^**IK), STAT2 (K**pY^690^**LK), STAT3 (P**pY^705^**LK), STAT4 (G**pY^693^**VP), STAT5A (G**pY^694^**VK), STAT5B (G**pY^699^**VK) and STAT6 (G**pY^641^**VP). Docking simulations for all STATs were carried out with Surflex-Dock 2.6 software [57] based on the pgeomx algorithm recommended for detailed studies of relative alignments. Exhaustive and time-consuming docking accuracy parameter set for optimal pose prediction of the compounds was used (density of search for specific spin alignment method set to 0.9, pre-dock and post-dock minimization, six additional starting conformations, minimum RMSD of 0.05 between poses, max 20 poses/ligand). As a result we obtained twenty binding poses of each structure in the predefined area of STAT-SH2 domain. Then, the best of twenty binding poses for each compound were filtered out for further analysis. By using the ‘STAT3-comparative binding affinity value’ (STAT3-CBAV) the binding quality between hSTAT3 and all the other STATs was compared for each compound. Finally, graphical presentation, measured by ‘ligand binding pose variation’ (LBPV) parameter of the results for STAT3-specific inhibitors was also validated. It was used to investigate the docking accuracy. LBPV described the ratio of conformers that had the binding position with RMSD < 0.5Å to the top scored one to all 20 output conformations obtained from docking. This parameter was adapted to the variety of inhibitory binding possibilities: LBPV_0_—conformational tendency towards pY+0, LBPV_X_—preference to fit in pY-X, LBPV_0+X_—binding to both cavities simultaneously.

### Comparative virtual screening of small compound libraries

First, the AMBER ff99SB charges were applied to all models of human STAT proteins. Small compounds from ZINC libraries were downloaded with ready-to-dock parameters of protonation state and partial atomic charges [58]. In virtual screening the same ligand-based approach, as in comparative docking, was used to generate a ‘protomol’. A five-step virtual screening procedure was employed to select the top STAT1 and STAT3 inhibitors. It was divided in the following steps:

1. *Pre-screen*

Docking simulations were carried out with Surflex-Dock 2.6 software [57] based on the pscreen algorithm recommended for large databases with fast screening parameter set (pre-dock minimization, post-dock minimization, max 3 poses/ligand). As a result we obtained three binding poses of each compound in the predefined area of STAT-SH2 domain. Additionally, each binding pose was supplied with the total score value representing the binding affinity of the compound to the SH2 domain.

2. *Primary filtering of inhibitors*

At first, the best of three binding poses for each compound were filtered out for further analysis. Then by using the STAT-CBAV the binding quality between different STATs was compared for each compound. Compounds with CBAV (for the STAT protein of interest) ≥ 3.0 were selected to re-screen (approx. 2000–3000 structures). Compounds with STAT-CBAV < 3.0 were removed from further analysis.

3. *Re-screen*

Repeated docking simulations were carried out with Surflex-Dock 2.6 software based on the pgeomx algorithm recommended for detailed studies of relative alignments. More exhaustive and time-consuming docking accuracy parameter set for optimal pose prediction of the compounds from primary filtering was used (density of search for specific spin alignment method set to 0.9, pre-dock and post-dock minimization, six additional starting conformations, minimum RMSD of 0.05 between poses, max 20 poses/ligand). As a result we obtained twenty binding poses of each compound in the predefined area of STAT-SH2 domain.

4. *Secondary filtering of inhibitors*

At first, the best of twenty binding poses for each compound were filtered out for further analysis. Then by using STAT-CBAV the binding quality between different STATs was compared for each compound. Compounds with CBAV (for the STAT protein of interest) ≥ 3.0 were selected to graphical validation (approx. 50–100 structures).

5. *Binding diversity of conformers*

Finally, graphical presentation, measured by LBPV parameter of the results for top 50–100 compounds was also validated. LBPV in range [0.8;1.0] represented low conformer diversity and very good binding specificity of the compound to STAT-SH2, whereas in range [0.0;0.2] denoted high conformer diversity and poor binding specificity.

Our tool combines CBAV (≥ 3.0) and LBPV (≥ 0.8 for STAT-specific and ≤ 0.2 STAT-non-specific) criteria to select the most specific STAT-SH2 targeting compounds.

## Results

### Homology modeling of human STAT monomers with their specific pTyr-linkers

**Fig 1:**
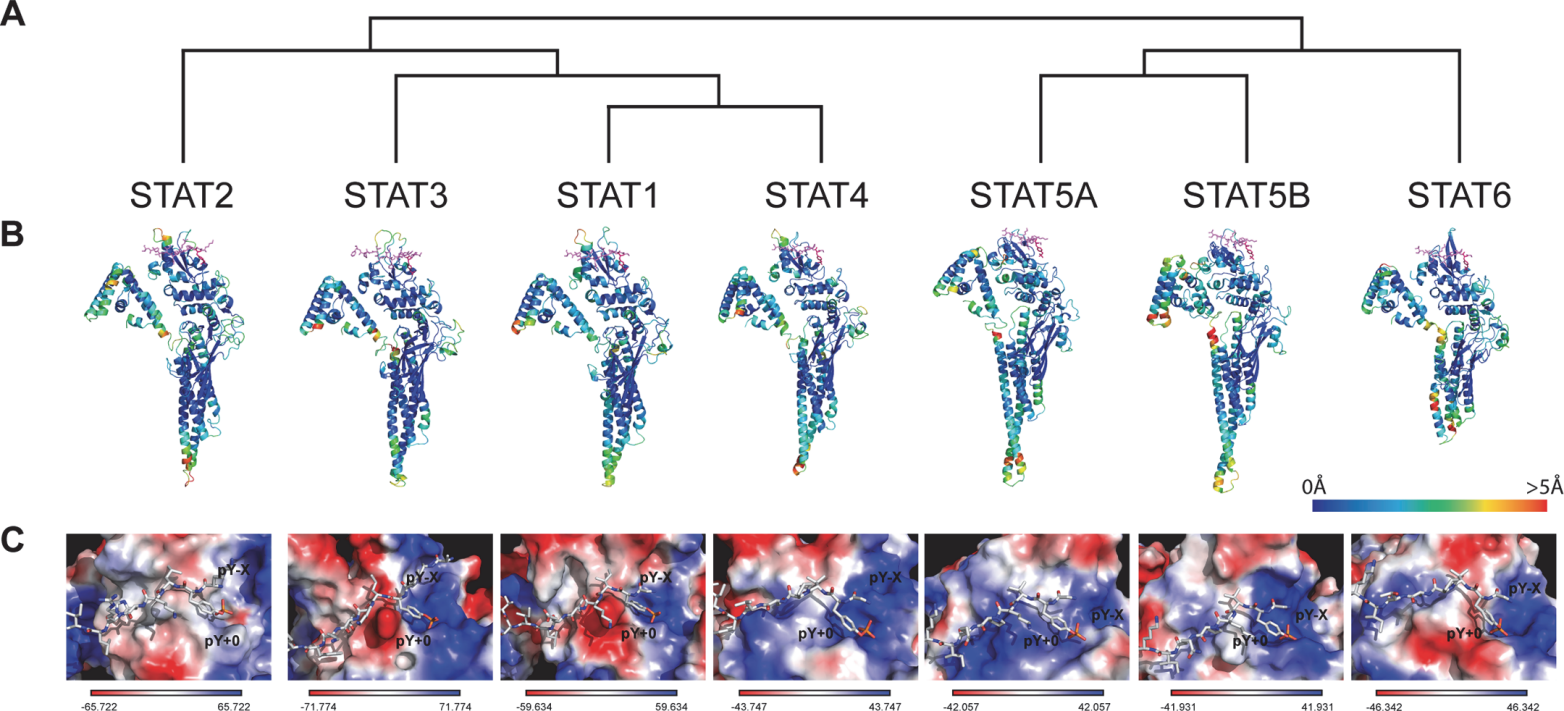
Structural models and phylogenetic comparison of hSTAT monomers (1, 2, 3, 4, 5A, 5B and 6) with their specific pTyr-linkers. **(A)** Phylogenetic distribution of hSTATs in form of a simplified phylogenetic tree. **(B)** Models of the monomers are shown in the cartoon representation with pTyr-peptides in the stick representation. Specific domains are positioned as follows: N-domain on the top-left, coiled-coiled domain on the bottom-center, C-domain on the top-right and SH2 domain on the top-center, to facilitate visual analysis of phosphotyrosine (pTyr)-linker and the SH2 interactions. Monomers are colored according to the predicted local deviation from the real structure (the predicted error of the model), as calculated by MetaMQAP. Blue indicates low predicted deviation of Cα atoms down to 0Å, red indicates unreliable regions with deviation > 5Å, green to orange indicate intermediate values. pTyr-peptides are colored in violet, while pTyr residue is colored in pink. **(C)** Models of hSTAT dimers with the linker of monomer I in the SH2 domain of monomer II. pTyr-peptides are presented in stick representation, pY+0—pTyr binding pocket, pY-X—hydrophobic side-pocket. SH2 domains are in the surface representation, colored according to the distribution of the electrostatic surface potential, calculated with APBS. Blue indicates positively charged regions, red indicates negatively charged regions.

To generate new 3D structure models for all hSTATs, we applied several methodologies. First, the most reliable structure and position of the hSTAT1, hSTAT2, hSTAT3, hSTAT4, hSTAT5A, hSTAT5B and hSTAT6 SH2 domain and pTyr-linker interaction site was determined, with respect to their complete protein structure. We performed multiple sequence alignment (MSA) of STAT sequences for vertebrates and phylogenetic analysis, presented in Fig. 1A in form of a simplified phylogenetic tree. Fig. 1A revealed that the STAT family could be subdivided into two groups. A large group of vertebrate sequences was represented by STAT1, STAT2, STAT3 and STAT4, while a smaller group only by STAT5A, STAT5B and STAT6.

Although crystal structures of human STAT1, murine STAT3 and STAT5A are available in the literature [11,28–30], the derived amino acid sequences of these crystal structures are not complete. Therefore, we decided to build our own models of all human STATs (see Methods and **S1 Fig.**). We built complete models of hSTAT1, by creating a hybrid structure of the 1YVL [28] and 1BF5 [29]; of hSTAT3—based on *M*. *musculus* 1BG1 [11] and *H*. *sapiens* 1YVL [28] and of hSTAT5A—based on *M*. *musculus* 1Y1U [30] and *H*. *sapiens* 1YVL [28] (**Fig. 1B and 1C**). Because the crystal structures of hSTAT2, hSTAT4, hSTAT5B and hSTAT6 have not been solved to date, a homology model of the *H*. *sapiens* counterpart of these STAT proteins as well as their corresponding pTyr-peptide fragment were built (see Methods) and we are the first to present them (**Fig. 1B and 1C**). **S1 Table** demonstrates scores of the chosen template structures for hSTATs, calculated by methods of protein homology detection, which are implemented at GeneSilico Metaserver. Based on the MetaMQAP and PROQ, assessment scores varied between 2.6Å and 3.0Å for RMSD and from 3.292 to 4.522 for LGscore (**Table 1**).

**Table 1:** Global accuracy scores of human STAT models.

Parameter	STAT1	STAT2	STAT3	STAT4	STAT5A	STAT5B	STAT6
*GDT_TS*	65.154	64.118	64.862	62.999	62.029	59.532	59.509
*RMSD [Å]*	2.5	2.6	2.6	2.7	2.7	3.0	2.8
*ProQ LGscore*	4.359	4.194	4.522	4.343	3.292	3.895	3.875
*ProQ MaxSub*	0.357	0.328	0.342	0.337	0.214	0.324	0.285
*Total Energy [kcal/mol]*	-5488.4	-4871.3	-5042.9	-4080.1	-5423.1	-5101.3	-3826.5

Graphical presentation of the SH2 domain for individual STATs, predicted the presence of the pY+0, pTyr-binding pocket and the hydrophobic side-pocket, pY-X [59] (**Fig. 1C and S2 Fig.**). Active residues (‘hot-spots’) identified by Park and Li [12] in hSTAT3-SH2, including Lys, Arg, Ser, Glu, Ser, appeared also present in the SH2 domain of other hSTATs (**S2 Table**). Structural superimposition of the pY+0 cavities for all hSTAT models [59] revealed that these ‘hot spots’ contain mostly conserved amino acids, with some group substitutions present in hSTAT2 (Arg instead of Lys) and STAT5A, STAT5B and STAT6 (Asp instead of Glu). Structural superimposition of the pY-X cavities for all hSTAT models [59] also showed high conservation of these residues (**S2 Table**), with group substitutions in hSTAT2 (Val instead of Ile), hSTAT4 (Val instead of Ile), hSTAT5A (Leu instead of Met, Val instead of Ile, Asn instead of Ser), hSTAT5B (Leu instead of Met, Val instead of Ile, Asn instead of Ser) and hSTAT6 (Ile instead of Met).

We also compared the arrangement of electrostatic potential of the protein surface among all hSTAT-SH2 models (**Fig. 1C and S2 Fig.**). Red color indicates negatively charged regions, determined by the presence of amino acids with negatively charged side chains, like Glu or Asp in pY+0. Blue color indicates positively charged regions with presence of positive amino acids e.g. Arg or Lys in pY+0. White color determines neutral regions, where amino acids have no electrostatic charge (e.g. hydrophobic Met, Phe, Gly and Val in pY-X). Comparing the electrostatic potential of the hSTAT-SH2 domains, combined with MSA [59], implies that pY+0 is positively charged in all hSTATs (**Fig. 1C and S2 Fig.**), and predicts that minor differences exist in amino acid composition. The pY-X region on the other hand is more divergent between different STATs. It is mostly neutral in case of hSTAT1; positively charged in case of hSTAT2, hSTAT4, hSTAT5A, hSTAT6; positive-negative in hSTAT3; positive-neutral in STAT5B. Together, differences in electrostatic potential of hSTAT-SH2 domains reflect minor differences in both pY+0 and pY-X cavities, which correspond to the evolutionary divergence of pTyr-linkers [59]. According to this homology analysis we also predicted the interaction sites between SH2 and pTyr linkers for all STATs (**Fig. 1C and S2 Fig.**). Based on this, we decided to use both binding pockets (pY+0 and pY-X) of all hSTATs as ‘protomols’ for subsequent virtual screening methods.

### Model validation by comparative docking of stattic to the SH2 domain of all hSTATs

In order to verify our hSTAT models we decided to first examine the questionable binding specificity of the known hSTAT3 inhibitor stattic for the hSTAT3-SH2 domain [16], by using a comparative docking strategy (chemical structure of stattic is displayed in **S3 Fig.**). First, docking simulation of stattic in all hSTAT-SH2 domains, using pgeomx algorithm, resulted in a list of 20 optimized conformations (see Methods), with corresponding binding affinity score values for each individual hSTAT (not shown). Table 2 shows the top binding affinity scores of stattic for each STAT, ranging between 2.90 (for STAT4) and 4.65 (for STAT2). As becomes clear from the calculated ‘STAT3-comparative binding affinity value’ (STAT3-CBAV), stattic exhibits a similar binding affinity to the SH2 domain of all STATs. STAT3-CBAVs for each STAT were lower than one, suggesting hSTAT-SH2 cross-binding (Table 2). Strikingly the STAT3-CBAV for hSTAT1 is close to zero, reflecting high conservation between these two STATs—they share 50% of global amino acid sequence homology, according to pairwise sequence identity analysis [60], see also S4 Fig. This was also confirmed by evolutionary conservation analysis of amino acid positions in hSTAT1 and hSTAT3 monomers, based on the MSA and phylogenetic relations using ConSurf [45]. Especially high conservation can be noticed in SH2 domain and DNA binding domain (S5 Fig.), indicated in dark purple. In case of SH2 domain the most conserved place is the interaction site between phosphotyrosine and pY+0 binding pocket, also indicated in dark purple.

**Table 2:** Top binding affinity scores for stattic (-logK_**D**_) towards individual hSTAT-SH2 domains, with predicted STAT3-CBAVs and LBPVs in pY+0 and pY-X cavities, obtained using Surflex-Dock 2.6 program.

STAT model	Top binding affinity	STAT3-CBAV	LBPV	
			pY+0	pY-X
*STAT1*	3.83	-0.04	0.55	0.45
*STAT2*	4.65	-0.86	0.85	0.15
*STAT3*	3.79	0.00	0.7	0.3
*STAT4*	2.90	0.89	1	0
*STAT5A*	3.53	0.26	1	0
*STAT5B*	3.64	0.15	0.1	0.9
*STAT6*	3.94	-0.15	0.8	0.2

**Fig 2:**
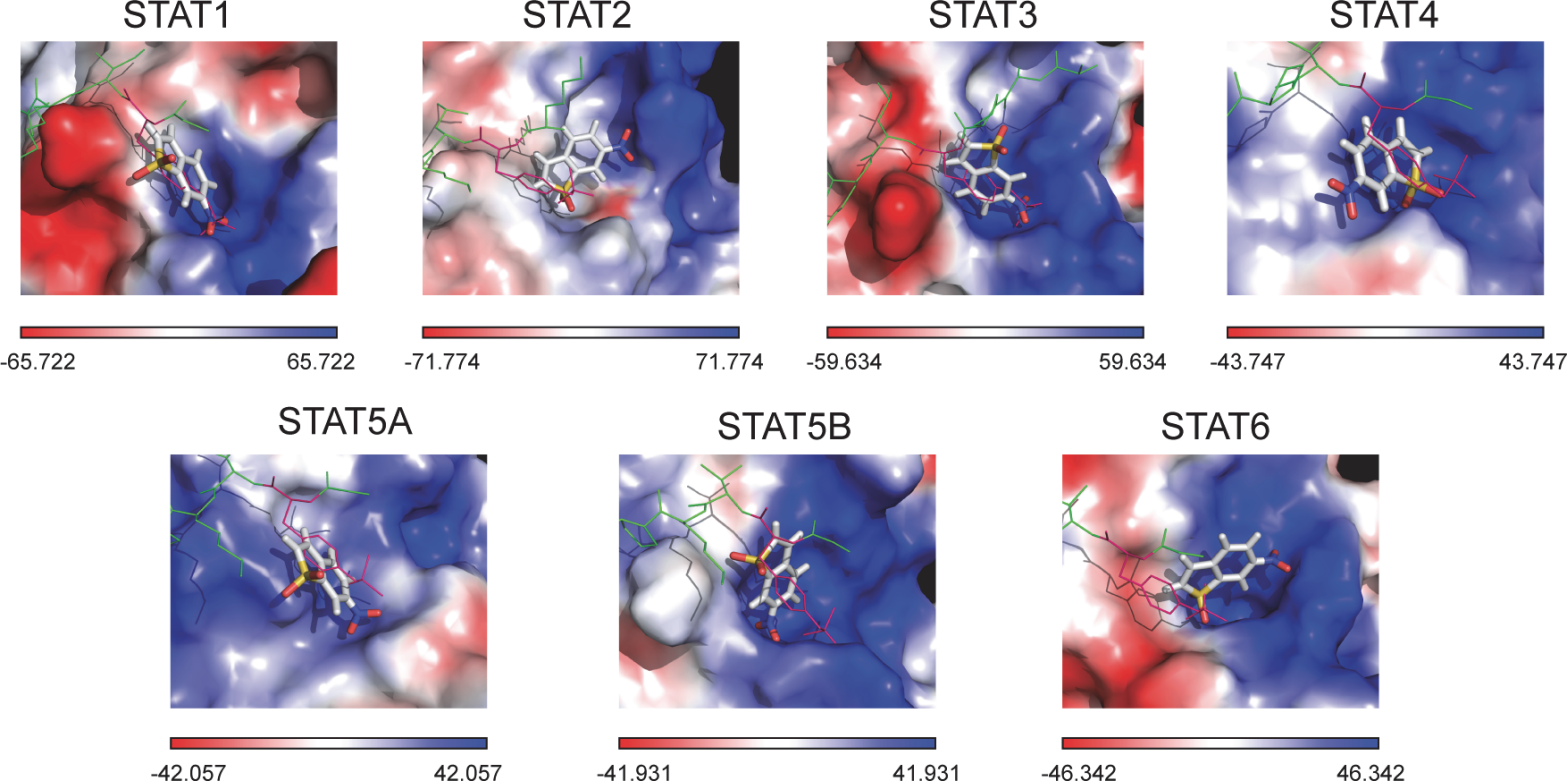
Top-scored binding conformation of stattic in the SH2 domain of all hSTATs. Stattic is shown in stick representation, pTyr-linker is presented as green colored lines with pTyr residue in pink. Results were obtained using Surflex-Dock 2.6 program.

In addition, we determined the ‘ligand binding pose variation’ (LBPV, see Methods) of stattic towards the hSTAT-SH2 pY+0 and pY-X cavities (LBPV_0_ indicates conformational tendency towards pY+0, LBPV_X_ towards pY-X, whereas LBPV_0+X_ towards both cavities simultaneously). We were able to calculate the conformational tendency of stattic to the hSTAT3-SH2. According to Table 2, from the top 20 optimized binding conformations of stattic to hSTAT3-SH2, 14 (70%) favor pY+0 and 6 (30%) fit to pY-X. LBPV analyses for other hSTAT-SH2 revealed that stattic also shares partial affinity between pY+0 and pY-X in case of hSTAT1, hSTAT2, hSTAT5B and hSTAT6 (Table 2) similar to hSTAT3. In contrast, for hSTAT4 and hSTAT5A stattic only fits in pY+0. These calculations were supported by graphical presentation of the docking results (Fig. 2) in which the top scored conformation of stattic for each individual STAT competes with pTyr in binding to the hSTAT-SH2 domain. Together, this supports the affirmation that due to its small size and low molecular weight stattic lacks STAT-SH2 binding specificity.

### hSTAT-comparative docking of selected STAT3-specific inhibitors

**Fig 3:**
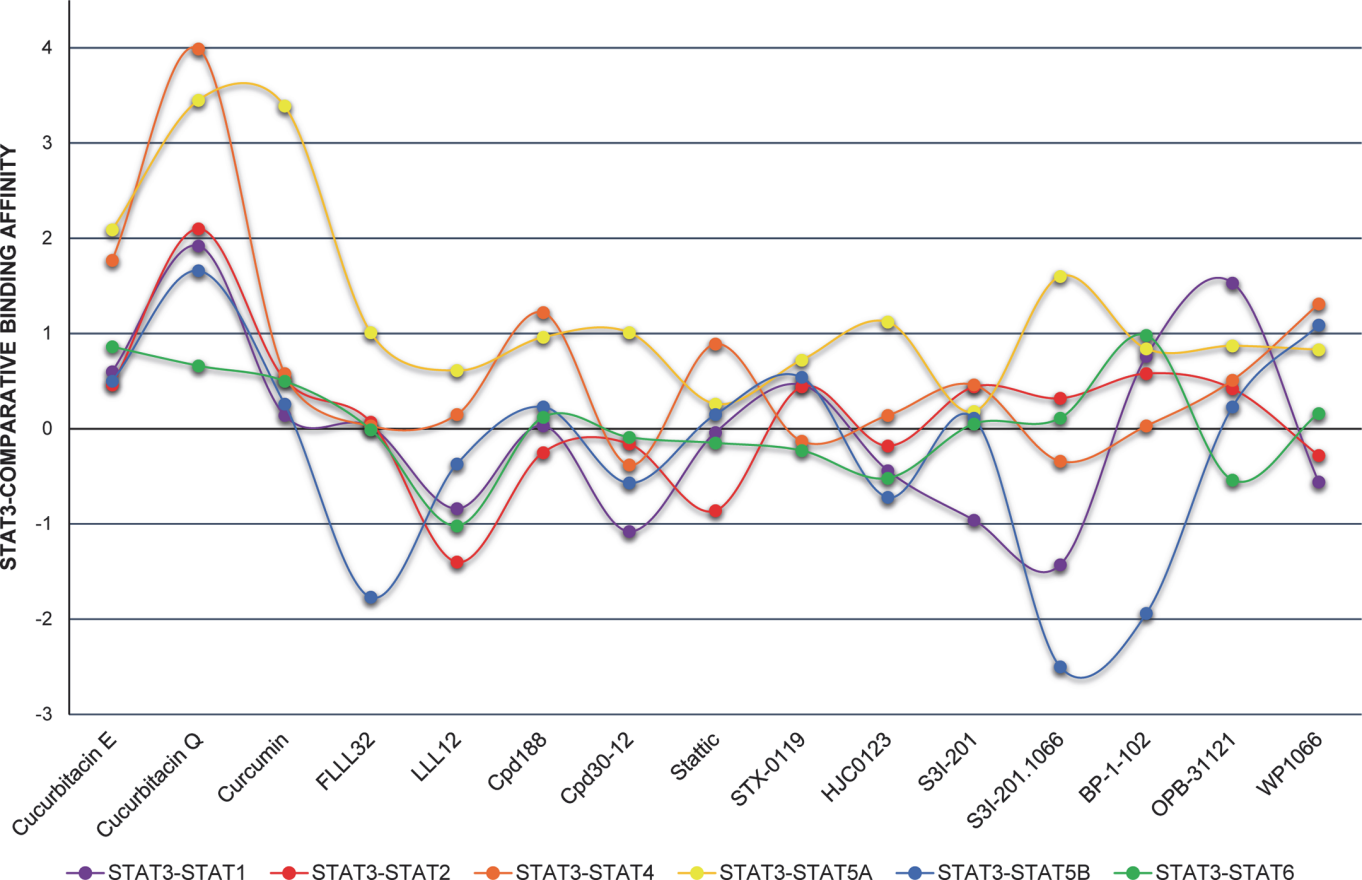
STAT3-CBAVs of STAT3-specific inhibitors. Graph presents comparative binding affinity values of a selection of STAT3-specific inhibitors docked to models of all hSTAT monomers.

This comparative docking strategy was subsequently applied to examine the binding specificity of a pre-selection of fourteen hSTAT3 inhibitors (**S3 Fig.**) for the hSTAT3-SH2 domain, as compared to that of all other hSTATs. As for stattic, docking simulations of these compounds in the hSTAT-SH2 domains resulted in a list of 20 optimized conformations (see Methods), with corresponding binding affinity score values for each individual hSTAT (not shown). **S3 Table** shows the top binding affinity scores of the individual STAT3 inhibitors for each STAT. STAT3 binding affinity values were the highest for natural compounds: cucurbitacin Q > curcumin > cucurbitacin E (9.08; 7.89; 7.49 respectively) (**S3 Table**). For the synthetic compounds, STAT3 binding affinities were lower than those for natural products with BP-1–102 and S3I-201.1066 exhibiting the highest ones (both 7.21) and LLL12 the lowest (3.95). Concerning binding affinity to all STATs, in general scores for stattic are the lowest [ranging from 2.9 (for STAT4) to 4.65 (for STAT2)] and for S3I-201.1066 the highest [ranging from 5.61 (for STAT5A) to 9.71 (for STAT5B)]. To obtain further insight into hSTAT-SH2 cross-binding specificity of these known STAT3 inhibitors, STAT3-CBAVs were calculated (**Fig. 3** and Table 3). According to **Fig. 3**, 74% of STAT3-CBAVs are between -1.0 and 1.0, while the majority of these compounds (94%) demonstrate STAT3-CBAVs between -2.0 and 2.0. This reflects a similar binding affinity of all of these compounds to the SH2 domain of individual STATs, as was seen with stattic. Only the natural compounds cucurbitacin Q (for STAT2 and STAT5A) and curcumin (for STAT5A) display STAT3-CBAVs higher than 3.0, which could point to a certain degree of STAT3 specificity.

**Table 3:** Predicted LBPVs of hSTAT3-specific inhibitors in pY+0 and pY-X cavities of hSTAT-SH2 domain, obtained using Surflex-Dock 2.6 program.

Compound	LBPV in pY+0 and/or pY-X													
	STAT1		STAT2		STAT3		STAT4		STAT5A		STAT5B		STAT6	
*BP-1–102*	0.45	0	0.65		1		0.6		0.9		0.8		0.4	
*Cpd188*	0.35		0.75		0.75		0.5		0.45	0	0.45	0.3	0.8	
*Cpd30–12*	0.65		0.8		0.65		0.35		0.65	0.2	0.75		0.45	
*Cucurbitacin E*	0.45		0.85		0.65		0.9		0.35	0.15	0.85		0.7	
*Cucurbitacin Q*	0.75		0.85		0.75		0.8	0	0.65	0	0.55		0.6	
*Curcumin*	0.75	0	0.65		0.65		0.6		0.4	0	0.8		0.35	
*FLLL32*	0.55		0.8		0.65		0.7		0.45	0	0.4		0.3	
*OPB-31121*	0.45	0	0.6		0.7		1	0	0.55		0	0.8	0.6	
*S3I-201*	0.5		0.75		0.7	0	0.7		0.4	0	0.3		0.3	
*S3I-201*.*1066*	0.45		0.5		0.6		0.55		0.35		0.7		0.6	0.4
*STX-0119*	0.5		0.35		0.55		0.8	0.2	0.6	0	0.4		0.6	
**HJC0123**	0.65	0	0.6	0.2	0.45	0.35	0.7	0.3	0.7	0	0.7		0.6	
**LLL12**	0.55		0.8		0.35	0.65	0.75	0	0.5	0	0.1	0.65	0.5	0
**Stattic**	0.55	0.45	0.85	0.15	0.7	0.3	1	0	1	0	0.1	0.9	0.8	0.2
**WP1066**	0.7	0.15	0.65		0	1	0.45	0	0.45	0	0.4		0.8	0

Single LBPV value represents binding of a specific compound to both cavities simultaneously. Double LBPV value represent situation, where compound binds to pY+0 and/or pY-X sub-pocket separately. Italic—compounds with predominant affinity to both pY+0 and pY-X sub-pockets; bold—compounds with predominant affinity to one of the pY+0 or pY-X sub-pockets.

We also calculated LBPVs for these fourteen compounds in hSTAT3 as compared to other hSTATs (**Table 3**, with stattic also included). For this purpose, the compounds were divided into two groups (see **Table 3**). The first group, labeled in italic, consists of complex and expanded compounds with high molecular weight and predominant affinity to both pY-0 and pY-X sub-pockets simultaneously (LBPV_0+X_). For example, cucurbitacin Q simultaneously binds to pY+0 and pY-X in STAT1, STAT2, STAT3, STAT5B and STAT6, with similar LBPV_0+X_ of 0.75, 0.85, 0.75, 0.55 and 0.6, respectively. In STAT4 and STAT5A, however, it only fits in pY+0 with LBPV_0_ of 0.8 and 0.65, respectively. For each individual compound in this group the LBPV for STAT3 is similar to LBPV for other STATs, correlating with hSTAT-SH2 cross-binding. Moreover, average LBPVs are lower than 0.8 and higher than 0.3, reflecting sub-optimal binding. The second group, labeled in bold, consists of small molecules like stattic with low molecular weight and predominant affinity to one of the pY+0 or pY-X sub-pockets (LBPV_0_ or LBPV_X_). For example, HJC0123 shares partial affinity between pY+0 and pY-X in case of hSTAT2, hSTAT3 and STAT4. In case of hSTAT1 and hSTAT5A, it only fits in pY+0. For hSTAT5B and hSTAT6, on the other hand, HJC0123 simultaneously binds pY+0 and pY-X (**Table 3**). When comparing the LBPV of STAT3 to that of other STATs, more variation is visible for this group of compounds, which could be due to their small size and low molecular weight and predicts lack of target specificity.

Based on the graphical presentation (**S6 Fig.**) we confirmed that in hSTAT3-SH2 these compounds primarily target the highly conserved pTyr-SH2 binding pocket (pY+0) and also highly conserved hydrophobic side-pocket (pY-X). From these results we conclude that none of these compounds are STAT3-specific, As such we propose that STAT3-CBAV ≤ 3.0 + LBPV_0+X_ ≤ 0.2 indicates STAT-cross-binding and STAT3-CBAV ≥ 3.0 + LBPV_0+X_ ≥ 0.8 predicts STAT-specificity, which is especially important for STAT1 and STAT3 cross-binding in aspect of their high SH2 domain homology (approximately 49% identity, see **S4 Fig.**).

### hSTAT comparative virtual screening of a small ligand library of natural products to identify STAT1 or STAT3-specific inhibitors

Based on the results obtained from the comparative docking of STAT3-specific inhibitors, we decided to apply a 5-step *in silico* hSTAT-SH2 comparative virtual screening strategy (for detailed description see Methods) for commercially available small compound libraries to identify specific STAT1 or STAT3 inhibitors. A ‘proof of principle’ test was performed on a ‘natural product’ library containing approx. 130 000 compounds. Virtual screening simulation of these compounds in the hSTAT-SH2 domain, using the pscreen algorithm, resulted in a list of 3 optimized conformations (see Methods) with supporting binding affinity score values (not shown) and successively STAT1-CBAVs and STAT3-CBAVs were calculated (not shown). After applying a threshold CBAV of ≥ 3.0, we obtained 215 top hits for hSTAT1 and 297 top hits for hSTAT3, which were used for the re-screen step (not shown), applying the pgeomx algorithm. This resulted in a list of 20 optimized conformations (see Methods) for each hSTAT, with supporting binding affinity values (not shown) and STAT1-CBAVs and STAT3-CBAVs. Accordingly, the top 5 potential specific inhibitors for hSTAT1 (**Table 4**) and hSTAT3 (**Table 5**) are presented, ordered by descending (STAT1-STAT3)-CBAVs and (STAT3-STAT1)-CBAVs, respectively. All these compounds exhibit high STAT1 or STAT3 binding affinity values (ranging between 11.0 and 15.0), whereas CBAV between STAT1 and STAT3 are in general above 3.5.

**Table 4:** Top hSTAT1-specific compounds selected from the natural products ZINC library, based on STAT1-CBAV and LBPV.

Compound ID	Top STAT1 binding affinity	STAT1-CBAV						LBPV_0+X_	
		STAT1-STAT2	STAT1-STAT3	STAT1-STAT4	STAT1-STAT5A	STAT1-STAT5B	STAT1-STAT6	STAT1	STAT3
*NP_1_1*	14.29	3.33	4.62	6.4	7.17	3.83	5.32	0.9	0
*NP_1_2*	11.43	4.72	3.76	5.68	4.78	3.84	3.58	1	0.35
*NP_1_3*	12.15	4.03	3.56	3.95	5.51	3.58	4.81	0.95	0.2
*NP_1_4*	14.89	7.21	3.53	6.78	6.9	6.47	5.98	0.9	0.15
*NP_1_5*	12.18	4.78	3.43	4.33	6.23	4.51	5.32	0.95	0.25

**Table 5:** Top hSTAT3-specific compounds selected from the natural products ZINC library, based on STAT3-CBAV and LBPV.

Compound ID	Top STAT3 binding affinity	STAT3-CBAV						LBPV_0+X_	
		STAT3-STAT1	STAT3-STAT2	STAT3-STAT4	STAT3-STAT5A	STAT3-STAT5B	STAT3-STAT6	STAT1	STAT3
*NP_3_1*	11.91	5.21	4.4	5.75	6.29	4.14	5.01	0.25	0.75
*NP_3_2*	14.34	5.2	4.15	6.95	7.25	3.59	7.3	0.15	0.9
*NP_3_3*	11.43	3.8	5.67	5.81	6.84	4.53	4.42	0.3	0.9
*NP_3_4*	12.95	3.79	4.25	5.38	6.39	3.27	6.17	0.25	0.9
*NP_3_5*	12.04	3.56	5.1	4.92	6.28	4.83	5.58	0.2	0.75

**Fig 4:**
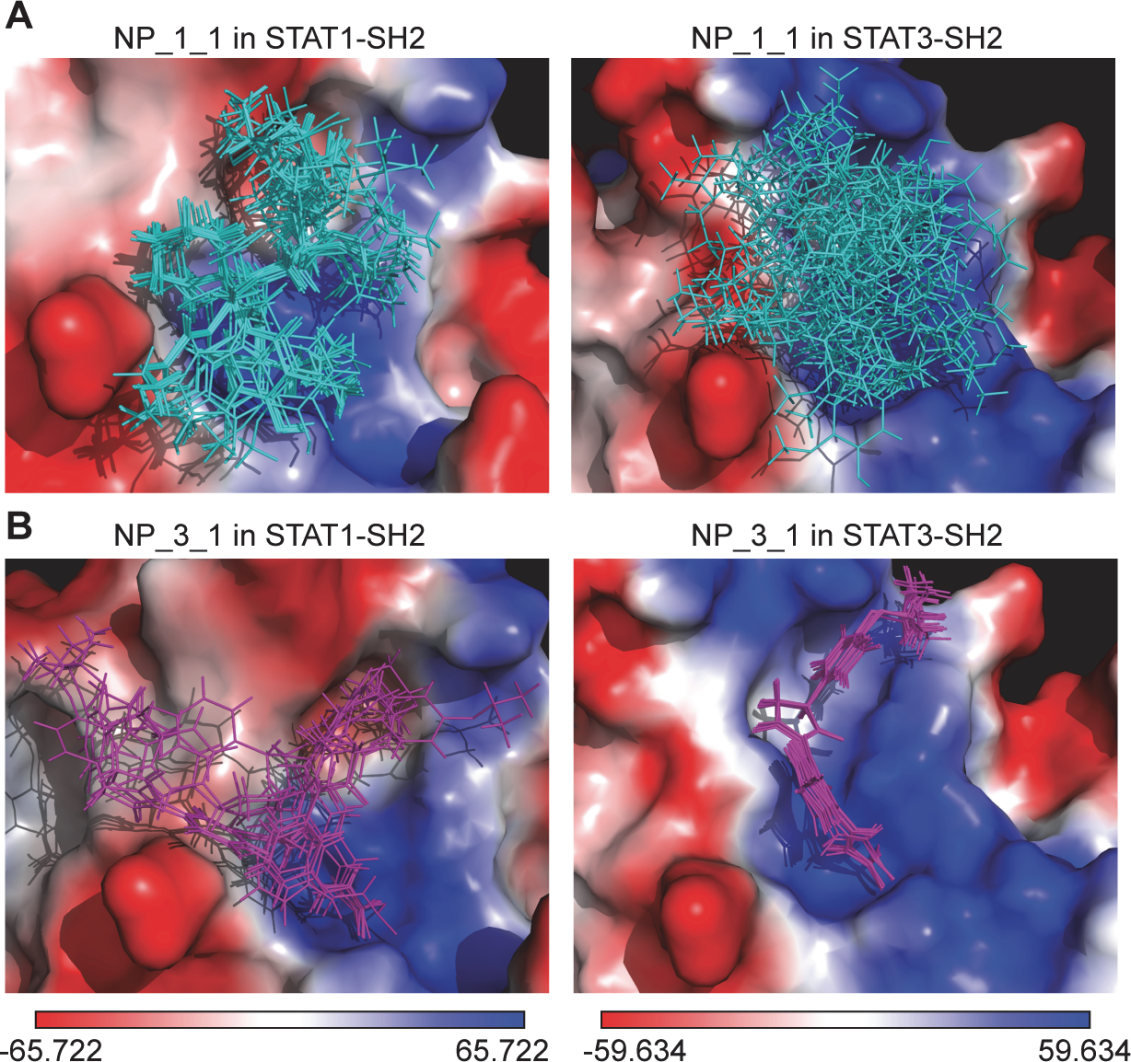
Binding conformations of top-scored compounds from natural products library in the SH2 domain of hSTAT1 and hSTAT3. **(A)** Binding pose variation of the top-scored hSTAT1-specific inhibitor in SH2 domain of hSTAT1 and hSTAT3. **(B)** Binding pose variation of the top-scored hSTAT3-specific inhibitor in SH2 domain of hSTAT1 and hSTAT3. The binding pose variations are shown in line representation, colored in blue and violet. Results were obtained using Surflex-Dock 2.6 program.

Additionally, LBPV_0+X_ for these compounds in hSTAT1-SH2 and hSTAT3-SH2 were calculated (Tables 4 and 5). As can be observed, the top 5 hSTAT1-specific compounds (NP_1_1-NP_1_5) display STAT1-LBPV_0+X_ ≥ 0.9 and STAT3-LBPV_0+X_ ≤ 0.35. In contrast, the top 5 hSTAT3-specific compounds (NP_3_1-NP_3_5 in **Table 5**) have STAT3-LBPV_0+X_ ≥ 0.75 while STAT1- LBPV_0+X_ is ≤ 0.3. This is further illustrated in **Fig. 4**, in which the top 20 optimized binding conformations for NP_1_1 (Fig. 4A) and NP_3_1 (Fig. 4B) are depicted in the SH2 domains of STAT1 and STAT3, as a graphical representation of LBPV_0+X_. As a representative hSTAT1 specific compound, with (STAT1-STAT3)-CBAV of 4.62, NP_1_1 has STAT1-LBPV_0+X_ of 0.9 and subsequent high conformational conservation within pY+0 and pY-X STAT1 sub-pockets. In STAT3-SH2, however, it also has predominant affinity to both pY+0 and pY-X but its STAT3-LBPV_0+X_ is only 0, which corresponds to no conformational conservation. Likewise, NP_3_1 displays high conformational conservation towards hSTAT3-SH2, but low conservation with respect to hSTAT1-SH2 (Fig. 4B).

### Application of comparative virtual screening pipeline approach in search of STAT1 or STAT3 specific inhibitors from multi-million ligand library of clean leads

Finally, a more advanced test of our *in silico* hSTAT-SH2 comparative virtual screening strategy was performed on a multi-million small compound clean leads library to identify specific STAT1 or STAT3 inhibitors. Docking of nearly 6 million compounds to the SH2 domain of each individual STAT was performed and a STAT1-CBAV and STAT3-CBAV threshold of ≥ 3.0 for all STATs was applied. Virtual screening simulation in the pscreen mode initially resulted in 1680 top hits for hSTAT1 and 1078 top hits for hSTAT3, which were used for the re-screen step (not shown). Based on a combination of 20 optimized conformations of each compound for individual hSTATs with supporting binding affinity values and STAT1-CBAVs and STAT3-CBAVs, the most promising compounds were selected. Thus, the top 5 potential specific inhibitors for hSTAT1 (Table 6) and hSTAT3 (Table 7) are presented, ordered by descending (STAT1-STAT3)-CBAVs and (STAT3-STAT1)-CBAVs, respectively. All these compounds exhibit high STAT1 or STAT3 binding affinity values (ranging between 9.0 and 11.0), whereas CBAV between STAT1 and STAT3 are in general above 4.5 for STAT1 inhibitors and above 3.0 for STAT3.

**Table 6:** Top hSTAT1-specific compounds selected from the clean leads ZINC library, based on STAT1-CBAV and LBPV.

Compound ID	Top STAT1 binding affinity	STAT1-CBAV						LBPV_0+X_	
		STAT1-STAT2	STAT1-STAT3	STAT1-STAT4	STAT1-STAT5A	STAT1-STAT5B	STAT1-STAT6	STAT1	STAT3
*CL_1_1*	10.36	4.84	5.47	6.32	5.14	4.66	5.15	0.8	0.1
*CL_1_2*	11.03	4.87	4.72	4.73	5.46	4.71	5.27	0.95	0.2
*CL_1_3*	10.46	3.97	4.67	4.99	5.56	4.52	4.84	0.8	0.15
*CL_1_4*	10.58	5.22	4.6	5.45	5.48	4.62	4.98	0.85	0.3
*CL_1_5*	10.65	4.88	4.54	3.95	5.37	4.44	4.42	0.75	0.2

**Table 7:** Top hSTAT3-specific compounds selected from the clean leads ZINC library, based on STAT3-CBAV and LBPV.

Compound ID	Top STAT3 binding affinity	STAT3-CBAV						LBPV_0+X_	
		STAT3-STAT1	STAT3-STAT2	STAT3-STAT4	STAT3-STAT5A	STAT3-STAT5B	STAT3-STAT6	STAT1	STAT3
*CL_3_1*	10.07	3.81	3.8	3.77	4.42	3.22	3.92	0.1	0.95
*CL_3_2*	9.33	3.76	3.09	3.39	4.57	3.26	3.2	0.15	0.75
*CL_3_3*	10.21	3.55	3.84	4.27	4.82	4.13	3.45	0.35	1
*CL_3_4*	10.15	3.51	3.6	3.14	4.81	3.82	3.94	0	0.9
*CL_3_5*	9.42	3.38	3.45	3.72	4.68	3.15	3.56	0.2	0.9

**Fig 5:**
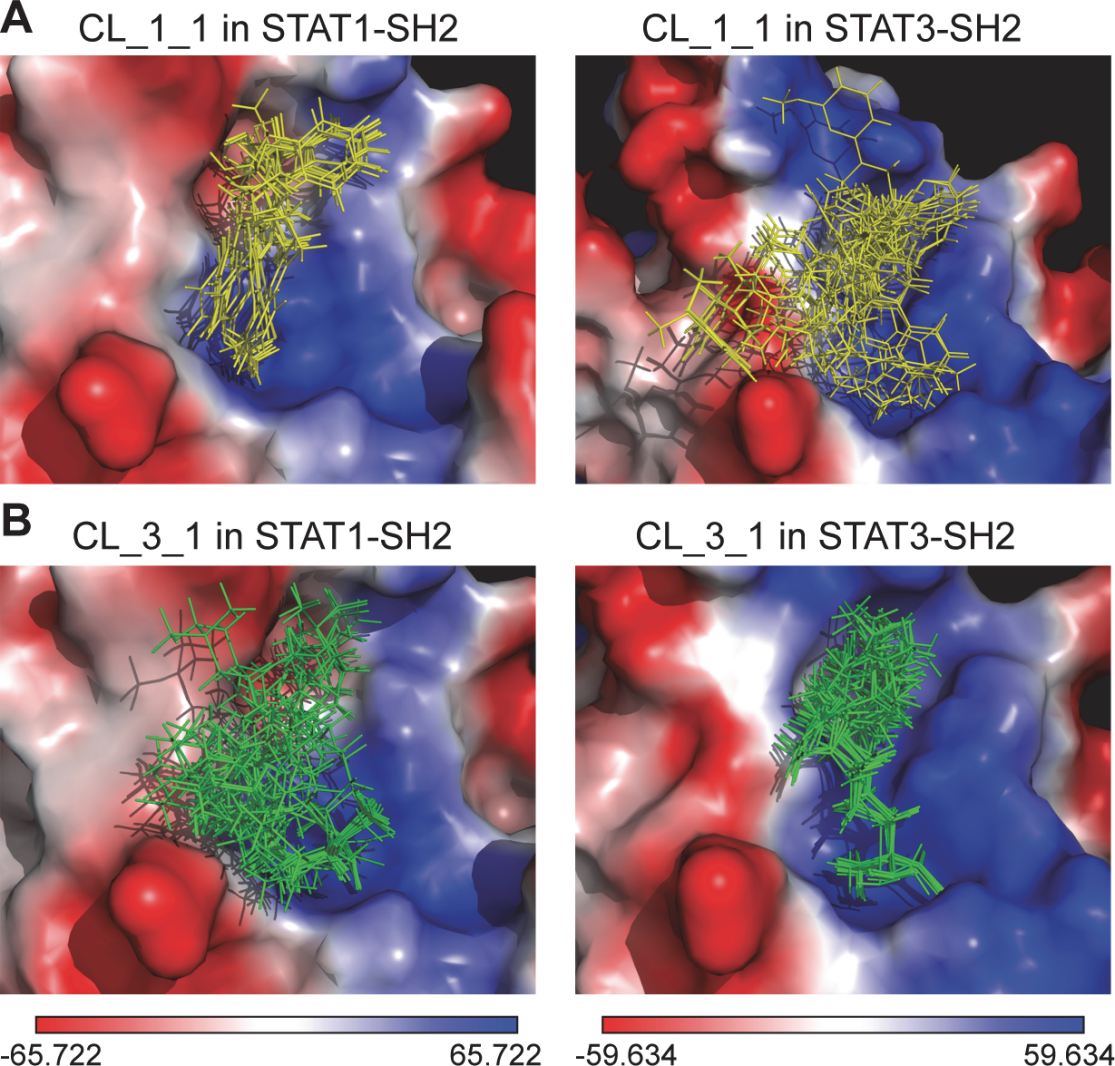
Binding conformations of top-scored compounds from clean leads library in the SH2 domain of hSTAT1 and hSTAT3. (A) Binding pose variation of the top-scored hSTAT1-specific inhibitor in SH2 domain of hSTAT1 and hSTAT3. (B) Binding pose variation of the top-scored hSTAT3-specific inhibitor in SH2 domain of hSTAT1 and hSTAT3. The binding pose variations are shown in line representation, colored in yellow and green. Results were obtained using Surflex-Dock 2.6 program.

Additionally, LBPV_0+X_ for these compounds in hSTAT1-SH2 and hSTAT3-SH2 were calculated (Tables 6 and 7). As can be observed, the top 5 hSTAT1-specific compounds (CL_1_1-CL_1_5 in Table 6) display STAT1-LBPV_0+X_ ≥ 0.75 and STAT3-LBPV_0+X_ ≤ 0.3. In contrast, the top 5 hSTAT3-specific compounds (CL_3_1-CL_3_5 in Table 7) have STAT3-LBPV_0+X_ ≥ 0.75 while STAT1- LBPV_0+X_ is ≤ 0.35. This is further illustrated in **Fig. 5**, in which the top 20 optimized binding conformations for CL_1_1 (Fig. 5A) and CL_3_1 (Fig. 5B) are depicted in the SH2 domains of STAT1 and STAT3, as a graphical representation of LBPV_0+X_. As a representative hSTAT1 specific compound, with (STAT1-STAT3)-CBAV of 5.47, CL_1_1 has STAT1-LBPV_0+X_ of 0.8 and subsequent high conformational conservation within pY+0 and pY-X STAT1 sub-pockets. In STAT3-SH2, however, it also has predominant affinity to both pY+0 and pY-X but its STAT3-LBPV_0+X_ is only 0,1, which corresponds to very low conformational conservation. Likewise, CL_3_1 displays high conformational conservation towards hSTAT3-SH2, but low conservation with respect to hSTAT1-SH2 (Fig. 5B).

## Discussion

Searches for STAT-targeting compounds, exploring the pTyr-SH2 interaction area, yielded many small molecules for STAT3 but sparsely for other STATs. Many of these inhibitors seem not STAT-specific, thereby questioning the present selection strategies of SH2 domain-based STAT inhibitors. This illustrates the need for better models, and screening and validation tools for more drugable STAT inhibitors with high specificity, potency and excellent bioavailability.

Therefore, our aim was to develop a novel bioinformatic selection strategy for STAT-specific inhibitory compounds using STAT-SH2 models in combination with comparative *in silico* virtual screening and docking validation.

First, we generated new 3D structure models for all human (h)STATs (1, 2, 3, 4, 5A, 5B and 6) based on multiple sequence alignment (MSA) of STAT vertebrate sequences and the limited crystal structures of STAT1, STAT3 and STAT5A [11,29,31]. Phylogenetic analysis confirmed close sequence and structural homology between all members of the STAT family, being subdivided into two groups (**Fig. 1A**); one consisting of STAT1, STAT2, STAT3 and STAT4, and the other of STAT5A, STAT5B and STAT6. In agreement with chromosomal localization of STAT genes (e.g. in *H*. *sapiens* STAT1 and STAT4—ch. 2, STAT3, STAT5A, STAT5B—ch. 17, STAT2 and STAT6—ch. 12) our analysis reflects the evolution of STATs through whole genome duplications and gene duplications by unequal chromosomal crossing-over [61]. Subsequently, by applying the homology modeling procedure, structural models of the *H*. *sapiens* counterparts of all STAT proteins as well as their corresponding pTyr-peptide fragment were built, with highly accurate final structural predictions (**Table** 1).

By focusing on the STAT-SH2 pTyr linker interaction, we studied in more detail the previously identified active residues in STAT3-SH2 [12]. We observed that the pY+0, pTyr-binding pocket and the hydrophobic side-pocket, pY-X, are highly conserved among all STATs [59] (**Fig. 1C and S2 Fig.**). Active residues (‘hot-spots’) identified by Park and Li [12] in hSTAT3-SH2, appeared also present in the SH2 domain of other hSTATs (**S2 Table**). Structural superimposition of the pY+0 and pY-X cavities for all hSTAT models [59] revealed that these ‘hot spots’ contain mostly conserved amino acids, and that their positions in the SH2 domain are fixed. This implicated high structural conservation of the pTyr-binding and hydrophobic pocket. However, structural superimposition of hSTAT-SH2 domains revealed the existence of additional lesser conserved regions in these cavities [59]. This observed divergence could be explained by the arrangement of electrostatic potential of the hSTAT-SH2 protein surface, which is clearly different among all STAT models (**Fig. 1C and S2 Fig.**). The superficial differences between hSTAT-SH2 of monomer I correspond to the evolutionary divergence of pTyr-linker from the monomer II (between all STATs). Based on the homology analysis we predicted the interactions between SH2 and pTyr linkers for all STATs (**Fig. 1C and S2 Fig.**). The mutual position of hSTAT-SH2 domain cavities and pTyr-peptide determines the specificity of dimer formation (**Fig. 1C and S2 Fig.**). This creates a possibility that by targeting the combination of pY+0 and pY-X sub-pockets in comparative virtual screening, involving all hSTAT models, will enable selection of STAT3 as well as STAT1-specific inhibitors.

Subsequently, a comparative *in silico* docking strategy was applied to obtain further insight into STAT-SH2 cross-binding specificity of the previously identified STAT3 inhibitor stattic. Stattic was reported to be a STAT3-specific (not STAT1 and STAT5) inhibitor [62]. It was proposed that stattic is a competitor of the phosphopeptide binding, which disrupts the STAT3-dimer formation. However, in 2012 Sanseverino et al. have shown, that in human monocyte-derived dendritic cells (MDDCs), stattic was able to reduce the level of IFNβ-induced STAT1 phosporylation and, to a lesser extent, of STAT2 phosphorylation [63]. Recently, we provided additional evidence that by primarily targeting the highly conserved pTyr-SH2 binding pocket stattic is not a specific hSTAT3 inhibitor, but is equally effective towards hSTAT1 and hSTAT2 [59]. This was confirmed in Human Micro-vascular Endothelial Cells (HMECs) *in vitro*, in which stattic inhibited interferon-α-induced phosphorylation of all three STATs. Here, by docking simulation of stattic in the SH2 domain of all hSTATs, combined with the STAT3-CBAV and LBPV parameters, we now further prove that stattic exhibits a similar binding affinity to the SH2 domain of all STATs by either targeting the pY+0 or pY-X cavity. Together, this supports the affirmation that due to its small size and low molecular weight stattic lacks STAT-SH2 binding specificity.

A similar docking approach was carried out to examine the binding specificity of a pre-selection of fourteen hSTAT3 inhibitors, including natural compounds (curcumin, cucurbitacin E and cucurbitacin Q) or chemical substances of synthetic origin (LLL12, FLLL32, Cpd188, Cpd30–12, STX-0119, HJC1023, S3I-201, S3I-201.1066, BP-1–102, OPB-31121 and WP1066), which were discovered by virtual screening or designed from previously described lead compounds to be hSTAT3-SH2 specific and fit in the functional cavities of hSTAT3-SH2 domain. Similar to stattic, the majority of these compounds primarily targeted the highly conserved pTyr-SH2 binding pocket of all STATs. Moreover, based on STAT3-CBAV and LBPV parameters and graphic representation in the SH2 domain of all hSTATs, we conclude that none of these compounds is STAT3-specific. Interestingly, smaller compounds, like HJC1023, LLL12, WP1066, were shown to predominantly target either the pY+0 or pY-X cavity, analogous to stattic. In contrast, compounds with higher molecular weight, including curcumin, cucurbitacin E, cucurbitacin Q, FLLL32, Cpd188, Cpd30–12, STX-0119, S3I-201, S3I-201.1066, BP-1–102, OPB-31121, covered both cavities for binding. Our comparative docking simulations correspond to the experimental studies of Bill et al. who proved the non-specificity of curcumin towards STAT3 and provided evidence of its cross-binding to STAT3 and STAT1 [64]. This also accounted for BP-1–102 [65], cryptotanshinone [66], Cpd30–12 [48], cyclopentenone derivatives [67], OPB-31121 [55], resveratrol analogs (RSVA314 and RSVA405) [68] and S3I-201 [7].

Docking simulation of the pre-selected hSTAT3 inhibitors in the SH2 domain of all hSTATs combined with proven cross-binding characteristics from *in vitro* experiments, also enabled us to identify STAT-CBAV and LBPV criteria correlating with ‘STAT cross-binding’ and ‘STAT-specificity’. Thus, we proposed that STAT3-CBAV ≤ 3.0 + LBPV_0+X_ ≤ 0.2 indicated STAT-cross-binding and STAT3-CBAV ≥ 3.0 + LBPV_0+X_ ≥ 0.8 predicted STAT-specificity. Based on these criteria we developed a novel *in silico* hSTAT-SH2 comparative virtual screening and docking validation strategy for commercially available small compound libraries to identify specific STAT inhibitors. Indeed, screening of a natural product library as well as a multi-million clean leads compound library successfully identified STAT1 as well as STAT3-specific inhibitors. For example, clean lead compound CL_1_1 was identified as hSTAT1-specific based on the combined criteria of (STAT1-STAT3)-CBAV of 5.47, STAT1-LBPV_0+X_ of 0.8 and STAT3-LBPV_0+X_ of 0.1. Consequently, CL_1_1 displayed high conformational conservation towards hSTAT1-SH2, but low conservation with respect to hSTAT3-SH2, which was confirmed by graphical representation in the SH2 domains of STAT1 and STAT3 (**Fig. 5A**). Likewise, CL_3_1 displayed high conformational conservation towards hSTAT3-SH2, but low conservation with respect to hSTAT1-SH2 (**Fig. 5B**), and was identified as hSTAT3-specific. Our novel screening approach highlights the great potential of the use of *in silico* hSTAT-SH2 comparative virtual screening and docking validation to identify specific STAT inhibitors. However, we realize that *in vitro* validation of STAT phosphorylation or STAT DNA-binding affinity is required to provide final proof of the value of our *in silico* screening tool. In this respect, following a simplified screening protocol (using only the pY+0 binding pocket of hSTAT1-SH2 as the protomol, combined with the pgeom algorithm in Surflex-Dock 2.6) we recently identified several new STAT1 inhibitors that after *in vitro* validation inhibited STAT1 phosphorylation but were also shown to inhibit STAT3 activity.

This is in agreement with the fact that identifying STAT-specific inhibitors meets many obstacles, from insufficient structural data to undefined mechanism of action, and lack of STAT-specificity of selected compounds. In-between there are also challenges with virtual and experimental validation and selection methods, like no uniformed screening protocols describing STAT-inhibitor binding affinities, not using the same STAT inducer for comparative *in vitro* assays, or lack of known inhibitors used as a reference point [7,64].

Therefore, based on our newly developed 3D structure models for all human STATs, we propose a standardized approach that combines comparative *in silico* virtual screening and docking validation of STAT-SH2 models with an *in vitro* multiple STAT phosphorylation assay, as a novel tool to screen multi-million clean lead-like and drug-like compound libraries and identify specific inhibitors for different STATs. ‘Specific’ STAT inhibitory compounds are subsequently selected based on the highest ‘comparative STAT binding affinity value’ combined with the highest conformational conservation. In an additional step a low throughput *in vitro* cell-based multiple STAT activation assay should be performed to test the effect of pre-selected inhibitory compounds on cytokine-induced and/or constitutive STAT phosphorylation in different cell types, following each *in silico* comparative screen [69]. Furthermore, by building the full-length hSTAT models, comparative *in silico* virtual screening can be combined with the comparative search for alternative STAT-specific inhibitor-binding cavities on the surface of STAT proteins (other than STAT-SH2 domain) and yield novel STAT-selective inhibitory strategies.

Development of an effective STAT inhibitor screening tool benefits the clinical need for more drugable STAT inhibitors. Identification of specific and effective STAT inhibitory compounds could provide a tool to increase our understanding of the functional role of STATs in different diseases, and could serve as therapeutic strategies in cancer, inflammation and auto-immunity. Although STATs represent highly attractive therapeutical targets for these diseases, so far no FDA approved treatment exists involving direct targeting of STAT proteins [10]. Therefore, the search for new STAT inhibitors with high specificity, potency and excellent bioavailability remains extremely important.

## Supporting Information

S1 Fig hSTATs—domains, homology models and templates. **SEQ:** full-length sequences with domains, (green)—**N:** N-terminal domain, **CC:** Coiled-coil domain, **DNA:** DNA-binding domain, **LK:** linker domain, **SH2:** Src-homology 2 domain, **Y:** phosphotyrosyl tail segment, **TA:** transcriptional activation domain; **P:** phosphorylated tyrosine (pink); **MOD:** homology models (orange) with phosphopeptides (red); **PDB IDs:** modeling templates (blue-violet scale). Length of the structures corresponds to the number of amino acids.
(TIF)

S2 Fig Enhanced representation of hSTAT dimer models with the linker of monomer I in the SH2 domain of monomer II. pTyr-peptides are presented in stick representation, pY+0—pTyr binding pocket is encircled by yellow dashed line, pY-X—hydrophobic side-pocket is encircled by pink dashed line. SH2 domains are in the surface representation, colored according to the distribution of the electrostatic surface potential, calculated with APBS. Blue indicates positively charged regions, red indicates negatively charged regions.
(TIF)

S3 Fig Selected hSTAT3-specific inhibitors. (TIF)

S4 Fig hSTAT protein pairwise sequence identity comparison. **(A)** Global comparison of full-length sequences of hSTATs. **(B)** Local comparison of hSTAT-SH2 domains. Upper comparison presents identities—the number of identical amino acids between two STATs. Lower comparison reflects percent identity of hSTATs.
(TIF)

S5 Fig Evolutionary conservation of amino acid positions in hSTAT1 and hSTAT3 monomers. **(A)** Models of hSTAT1 and hSTAT3 monomers in the surface representation with pTyr-peptides in the stick representation. **(B)** Models of hSTAT1-SH2 and hSTAT3-SH2 domains in the surface representation with pTyr-linkers, shown as sticks. Structures are colored according to sequence similarity, based on the multiple sequence alignments and phylogenetic relations using ConSurf. Purple indicates conserved residues, white to blue indicate variable residues. pTyr-peptides are colored in green, while pTyr residue is colored in yellow.
(TIF)

S6 Fig Top-scored binding conformation of hSTAT3-specific inhibitors in the SH2 domain of hSTAT3. Inhibitors are shown in stick representation, pTyr-linker is presented as lines colored in green with pTyr residue colored in pink. Results were obtained using Surflex-Dock 2.6 program.
(TIF)

S1 Table Scores of the optimal template structures for hSTATs. Values were calculated by selected methods of protein homology detection, which are implemented at GeneSilico Metaserver; n/a—no result.
(DOCX)

S2 Table Amino acid ‘Hot spot’ analysis for the pY+0 and pY-X binding sub-pockets in the hSTAT-SH2 domains. Based on the multiple sequence alignment and hSTAT models superimposition. Bold—group substitutions of the amino acids in comparison to hSTAT1.
(DOCX)

S3 Table Binding affinities for hSTAT3-specific inhibitors, represented by top-scored conformers. Results were obtained using Surflex-Dock 2.6 program.
(DOCX)

## References

[1] Levy DE, Darnell JE Jr (2002) Stats: transcriptional control and biological impact. Nat Rev Mol Cell Biol 3: 651–662. 12209125 10.1038/nrm909

[2] Horvath CM (2000) STAT proteins and transcriptional responses to extracellular signals. Trends Biochem Sci 25: 496–502. 11050435 10.1016/s0968-0004(00)01624-8

[3] El Kasmi KC, Holst J, Coffre M, Mielke L, de Pauw A, et al (2006) General nature of the STAT3-activated anti-inflammatory response. J Immunol 177: 7880–7888. 17114459 10.4049/jimmunol.177.11.7880

[4] Kamran MZ, Patil P, Gude RP (2013) Role of STAT3 in cancer metastasis and translational advances. Biomed Res Int 2013: 421821 10.1155/2013/421821 24199193 PMC3807846

[5] Sikorski K, Czerwoniec A, Bujnicki JM, Wesoly J, Bluyssen HA (2011) STAT1 as a novel therapeutical target in pro-atherogenic signal integration of IFNgamma, TLR4 and IL-6 in vascular disease. Cytokine Growth Factor Rev 22: 211–219. 10.1016/j.cytogfr.2011.06.003 21752694

[6] Yu H, Pardoll D, Jove R (2009) STATs in cancer inflammation and immunity: a leading role for STAT3. Nat Rev Cancer 9: 798–809. 10.1038/nrc2734 19851315 PMC4856025

[7] Debnath B, Xu S, Neamati N (2012) Small molecule inhibitors of signal transducer and activator of transcription 3 (Stat3) protein. J Med Chem 55: 6645–6668. 10.1021/jm300207s 22650325

[8] Furqan M, Akinleye A, Mukhi N, Mittal V, Chen Y, et al (2013) STAT inhibitors for cancer therapy. J Hematol Oncol 6: 90 10.1186/1756-8722-6-90 24308725 PMC4029528

[9] Ma DL, Liu LJ, Leung KH, Chen YT, Zhong HJ, et al (2014) Antagonizing STAT3 dimerization with a rhodium(III) complex. Angew Chem Int Ed Engl 53: 9178–9182. 10.1002/anie.201404686 24889897

[10] Miklossy G, Hilliard TS, Turkson J (2013) Therapeutic modulators of STAT signalling for human diseases. Nat Rev Drug Discov 12: 611–629. 10.1038/nrd4088 23903221 PMC4038293

[11] Becker S, Groner B, Muller CW (1998) Three-dimensional structure of the Stat3beta homodimer bound to DNA. Nature 394: 145–151. 9671298 10.1038/28101

[12] Park IH, Li C (2011) Characterization of molecular recognition of STAT3 SH2 domain inhibitors through molecular simulation. J Mol Recognit 24: 254–265. 10.1002/jmr.1047 21360612

[13] Bromberg JF, Horvath CM, Besser D, Lathem WW, Darnell JE Jr (1998) Stat3 activation is required for cellular transformation by v-src. Mol Cell Biol 18: 2553–2558. 9566875 10.1128/mcb.18.5.2553 PMC110635

[14] Turkson J, Jove R (2000) STAT proteins: novel molecular targets for cancer drug discovery. Oncogene 19: 6613–6626. 11426647 10.1038/sj.onc.1204086

[15] Song H, Wang R, Wang S, Lin J (2005) A low-molecular-weight compound discovered through virtual database screening inhibits Stat3 function in breast cancer cells. Proc Natl Acad Sci U S A 102: 4700–4705. 15781862 10.1073/pnas.0409894102 PMC555708

[16] McMurray JS (2006) A new small-molecule Stat3 inhibitor. Chem Biol 13: 1123–1124. 17113993 10.1016/j.chembiol.2006.11.001

[17] Lin L, Hutzen B, Li PK, Ball S, Zuo M, et al (2010) A novel small molecule, LLL12, inhibits STAT3 phosphorylation and activities and exhibits potent growth-suppressive activity in human cancer cells. Neoplasia 12: 39–50. 20072652 10.1593/neo.91196 PMC2805882

[18] Shakibaei M, Harikumar KB, Aggarwal BB (2009) Resveratrol addiction: to die or not to die. Mol Nutr Food Res 53: 115–128. 10.1002/mnfr.200800148 19072742

[19] Tu SP, Jin H, Shi JD, Zhu LM, Suo Y, et al (2012) Curcumin induces the differentiation of myeloid-derived suppressor cells and inhibits their interaction with cancer cells and related tumor growth. Cancer Prev Res (Phila) 5: 205–215. 10.1158/1940-6207.CAPR-11-0247 22030090 PMC3273601

[20] Altschul SF, Gish W, Miller W, Myers EW, Lipman DJ (1990) Basic local alignment search tool. J Mol Biol 215: 403–410. 2231712 10.1016/S0022-2836(05)80360-2

[21] Edgar RC (2004) MUSCLE: multiple sequence alignment with high accuracy and high throughput. Nucleic Acids Res 32: 1792–1797. 15034147 10.1093/nar/gkh340 PMC390337

[22] Tamura K, Stecher G, Peterson D, Filipski A, Kumar S (2013) MEGA6: Molecular Evolutionary Genetics Analysis version 6.0. Mol Biol Evol 30: 2725–2729. 10.1093/molbev/mst197 24132122 PMC3840312

[23] Kurowski MA, Bujnicki JM (2003) GeneSilico protein structure prediction meta-server. Nucleic Acids Res 31: 3305–3307. 12824313 10.1093/nar/gkg557 PMC168964

[24] Soding J (2005) Protein homology detection by HMM-HMM comparison. Bioinformatics 21: 951–960. 15531603 10.1093/bioinformatics/bti125

[25] McGuffin LJ, Jones DT (2003) Improvement of the GenTHREADER method for genomic fold recognition. Bioinformatics 19: 874–881. 12724298 10.1093/bioinformatics/btg097

[26] Sadreyev RI, Baker D, Grishin NV (2003) Profile-profile comparisons by COMPASS predict intricate homologies between protein families. Protein Sci 12: 2262–2272. 14500884 10.1110/ps.03197403 PMC2366929

[27] Wallner B, Elofsson A (2005) Pcons5: combining consensus, structural evaluation and fold recognition scores. Bioinformatics 21: 4248–4254. 16204344 10.1093/bioinformatics/bti702

[28] Mao X, Ren Z, Parker GN, Sondermann H, Pastorello MA, et al (2005) Structural bases of unphosphorylated STAT1 association and receptor binding. Mol Cell 17: 761–771. 15780933 10.1016/j.molcel.2005.02.021

[29] Chen X, Vinkemeier U, Zhao Y, Jeruzalmi D, Darnell JE Jr., et al (1998) Crystal structure of a tyrosine phosphorylated STAT-1 dimer bound to DNA. Cell 93: 827–839. 9630226 10.1016/s0092-8674(00)81443-9

[30] Neculai D, Neculai AM, Verrier S, Straub K, Klumpp K, et al (2005) Structure of the unphosphorylated STAT5a dimer. J Biol Chem 280: 40782–40787. 16192273 10.1074/jbc.M507682200

[31] Sali A, Blundell TL (1993) Comparative protein modelling by satisfaction of spatial restraints. J Mol Biol 234: 779–815. 8254673 10.1006/jmbi.1993.1626

[32] Stewart JJP (1989) Optimization of Parameters for Semiempirical Methods. 1. Method. Journal of Computational Chemistry 10: 209–220.

[33] Frisch MJ, Trucks GW, Schlegel HB, Scuseria GE, Robb MA, et al (2009) Gaussian 09, Revision A.02 Gaussian, Inc, Wallingford, CT

[34] Pawlowski M, Gajda MJ, Matlak R, Bujnicki JM (2008) MetaMQAP: a meta-server for the quality assessment of protein models. BMC Bioinformatics 9: 403 10.1186/1471-2105-9-403 18823532 PMC2573893

[35] Boniecki M, Rotkiewicz P, Skolnick J, Kolinski A (2003) Protein fragment reconstruction using various modeling techniques. J Comput Aided Mol Des 17: 725–738. 15072433 10.1023/b:jcam.0000017486.83645.a0

[36] Hildebrand PW, Goede A, Bauer RA, Gruening B, Ismer J, et al (2009) SuperLooper—a prediction server for the modeling of loops in globular and membrane proteins. Nucleic Acids Res 37: W571–574. 10.1093/nar/gkp338 19429894 PMC2703960

[37] Ramachandran S, Kota P, Ding F, Dokholyan NV (2011) Automated minimization of steric clashes in protein structures. Proteins 79: 261–270. 10.1002/prot.22879 21058396 PMC3058769

[38] Wallner B, Elofsson A (2003) Can correct protein models be identified? Protein Sci 12: 1073–1086. 12717029 10.1110/ps.0236803 PMC2323877

[39] Carugo O, Pongor S (2001) A normalized root-mean-square distance for comparing protein three-dimensional structures. Protein Sci 10: 1470–1473. 11420449 10.1110/ps.690101 PMC2374114

[40] Larsson P, Skwark MJ, Wallner B, Elofsson A (2009) Assessment of global and local model quality in CASP8 using Pcons and ProQ. Proteins 77 Suppl 9: 167–172. 10.1002/prot.22476 19544566

[41] Read RJ, Chavali G (2007) Assessment of CASP7 predictions in the high accuracy template-based modeling category. Proteins 69 Suppl 8: 27–37. 17894351 10.1002/prot.21662

[42] Zemla A, Venclovas C, Moult J, Fidelis K (1999) Processing and analysis of CASP3 protein structure predictions. Proteins Suppl 3: 22–29. 10.1002/(sici)1097-0134(1999)37:3+<22::aid-prot5>3.3.co;2-n 10526349

[43] DeLano WL (2002) The PyMOL molecular graphics system San Carlos, CA: DeLano Scientific

[44] Baker NA, Sept D, Joseph S, Holst MJ, McCammon JA (2001) Electrostatics of nanosystems: application to microtubules and the ribosome. Proc Natl Acad Sci U S A 98: 10037–10041. 11517324 10.1073/pnas.181342398 PMC56910

[45] Glaser F, Pupko T, Paz I, Bell RE, Bechor-Shental D, et al (2003) ConSurf: identification of functional regions in proteins by surface-mapping of phylogenetic information. Bioinformatics 19: 163–164. 12499312 10.1093/bioinformatics/19.1.163

[46] Sun J, Blaskovich MA, Jove R, Livingston SK, Coppola D, et al (2005) Cucurbitacin Q: a selective STAT3 activation inhibitor with potent antitumor activity. Oncogene 24: 3236–3245. 15735720 10.1038/sj.onc.1208470

[47] Bharti AC, Donato N, Aggarwal BB (2003) Curcumin (diferuloylmethane) inhibits constitutive and IL-6-inducible STAT3 phosphorylation in human multiple myeloma cells. J Immunol 171: 3863–3871. 14500688 10.4049/jimmunol.171.7.3863

[48] Xu X, Kasembeli MM, Jiang X, Tweardy BJ, Tweardy DJ (2009) Chemical probes that competitively and selectively inhibit Stat3 activation. PLoS One 4: e4783 10.1371/journal.pone.0004783 19274102 PMC2653189

[49] Matsuno K, Masuda Y, Uehara Y, Sato H, Muroya A, et al (2010) Identification of a New Series of STAT3 Inhibitors by Virtual Screening. ACS Med Chem Lett 1: 371–375. 10.1021/ml1000273 24900220 PMC4007973

[50] Siddiquee K, Zhang S, Guida WC, Blaskovich MA, Greedy B, et al (2007) Selective chemical probe inhibitor of Stat3, identified through structure-based virtual screening, induces antitumor activity. Proc Natl Acad Sci U S A 104: 7391–7396. 17463090 10.1073/pnas.0609757104 PMC1863497

[51] Zhang X, Yue P, Fletcher S, Zhao W, Gunning PT, et al (2010) A novel small-molecule disrupts Stat3 SH2 domain-phosphotyrosine interactions and Stat3-dependent tumor processes. Biochem Pharmacol 79: 1398–1409. 10.1016/j.bcp.2010.01.001 20067773 PMC3188443

[52] Iwamaru A, Szymanski S, Iwado E, Aoki H, Yokoyama T, et al (2007) A novel inhibitor of the STAT3 pathway induces apoptosis in malignant glioma cells both in vitro and in vivo. Oncogene 26: 2435–2444. 17043651 10.1038/sj.onc.1210031

[53] Bill MA, Fuchs JR, Li C, Yui J, Bakan C, et al (2010) The small molecule curcumin analog FLLL32 induces apoptosis in melanoma cells via STAT3 inhibition and retains the cellular response to cytokines with anti-tumor activity. Mol Cancer 9: 165 10.1186/1476-4598-9-165 20576164 PMC2902420

[54] Chen H, Yang Z, Ding C, Chu L, Zhang Y, et al (2013) Fragment-based drug design and identification of HJC0123, a novel orally bioavailable STAT3 inhibitor for cancer therapy. Eur J Med Chem 62: 498–507. 10.1016/j.ejmech.2013.01.023 23416191 PMC3750725

[55] Kim MJ, Nam HJ, Kim HP, Han SW, Im SA, et al (2013) OPB-31121, a novel small molecular inhibitor, disrupts the JAK2/STAT3 pathway and exhibits an antitumor activity in gastric cancer cells. Cancer Lett 335: 145–152. 10.1016/j.canlet.2013.02.010 23402820

[56] Zhao Y, Truhlar DG (2008) The M06 suite of density functionals for main group thermochemistry, thermochemical kinetics, noncovalent interactions, excited states, and transition elements: two new functionals and systematic testing of four M06-class functionals and 12 other functionals. Theoretical Chemistry Accounts 120: 215–241.

[57] Jain AN (2003) Surflex: fully automatic flexible molecular docking using a molecular similarity-based search engine. J Med Chem 46: 499–511. 12570372 10.1021/jm020406h

[58] Irwin JJ, Shoichet BK (2005) ZINC—a free database of commercially available compounds for virtual screening. J Chem Inf Model 45: 177–182. 15667143 10.1021/ci049714 PMC1360656

[59] Szelag M, Sikorski K, Czerwoniec A, Szatkowska K, Wesoly J, et al (2013) In silico simulations of STAT1 and STAT3 inhibitors predict SH2 domain cross-binding specificity. Eur J Pharmacol 720: 38–48. 10.1016/j.ejphar.2013.10.055 24211327

[60] Lin J, Buettner R, Yuan YC, Yip R, Horne D, et al (2009) Molecular dynamics simulations of the conformational changes in signal transducers and activators of transcription, Stat1 and Stat3. J Mol Graph Model 28: 347–356. 10.1016/j.jmgm.2009.08.013 19781967

[61] Wang Y, Levy DE (2012) Comparative evolutionary genomics of the STAT family of transcription factors. JAKSTAT 1: 23–33. 10.4161/jkst.19418 24058748 PMC3670131

[62] Schust J, Sperl B, Hollis A, Mayer TU, Berg T (2006) Stattic: a small-molecule inhibitor of STAT3 activation and dimerization. Chem Biol 13: 1235–1242. 17114005 10.1016/j.chembiol.2006.09.018

[63] Sanseverino I, Purificato C, Gauzzi MC, Gessani S (2012) Revisiting the specificity of small molecule inhibitors: the example of stattic in dendritic cells. Chem Biol 19: 1213–1214; author reply 1215–1216. 10.1016/j.chembiol.2012.08.021 23102212

[64] Bill MA, Nicholas C, Mace TA, Etter JP, Li C, et al (2012) Structurally modified curcumin analogs inhibit STAT3 phosphorylation and promote apoptosis of human renal cell carcinoma and melanoma cell lines. PLoS One 7: e40724 10.1371/journal.pone.0040724 22899991 PMC3416819

[65] Zhang X, Yue P, Page BD, Li T, Zhao W, et al (2012) Orally bioavailable small-molecule inhibitor of transcription factor Stat3 regresses human breast and lung cancer xenografts. Proc Natl Acad Sci U S A 109: 9623–9628. 10.1073/pnas.1121606109 22623533 PMC3386073

[66] Shin DS, Kim HN, Shin KD, Yoon YJ, Kim SJ, et al (2009) Cryptotanshinone inhibits constitutive signal transducer and activator of transcription 3 function through blocking the dimerization in DU145 prostate cancer cells. Cancer Res 69: 193–202. 10.1158/0008-5472.CAN-08-2575 19118003

[67] Gunning PT, Katt WP, Glenn M, Siddiquee K, Kim JS, et al (2007) Isoform selective inhibition of STAT1 or STAT3 homo-dimerization via peptidomimetic probes: structural recognition of STAT SH2 domains. Bioorg Med Chem Lett 17: 1875–1878. 17336521 10.1016/j.bmcl.2007.01.077

[68] Capiralla H, Vingtdeux V, Zhao H, Sankowski R, Al-Abed Y, et al (2012) Resveratrol mitigates lipopolysaccharide- and Abeta-mediated microglial inflammation by inhibiting the TLR4/NF-kappaB/STAT signaling cascade. J Neurochem 120: 461–472. 10.1111/j.1471-4159.2011.07594.x 22118570 PMC3253186

[69] Szelag M, Czerwoniec A, Wesoly J, Bluyssen HA (2014) Comparative screening and validation as a novel tool to identify STAT-specific inhibitors. Eur J Pharmacol 740: 417–420. 10.1016/j.ejphar.2014.05.047 25183399

