# Supplementary figures and images for: Identification of STAT1 and STAT3 Specific Inhibitors Using Comparative Virtual Screening and Docking Validation
Source: PLoS One. 2015 Feb 24;10(2):e0116688. doi: 10.1371/journal.pone.0116688 (PMC4339377; doi:10.1371/journal.pone.0116688)

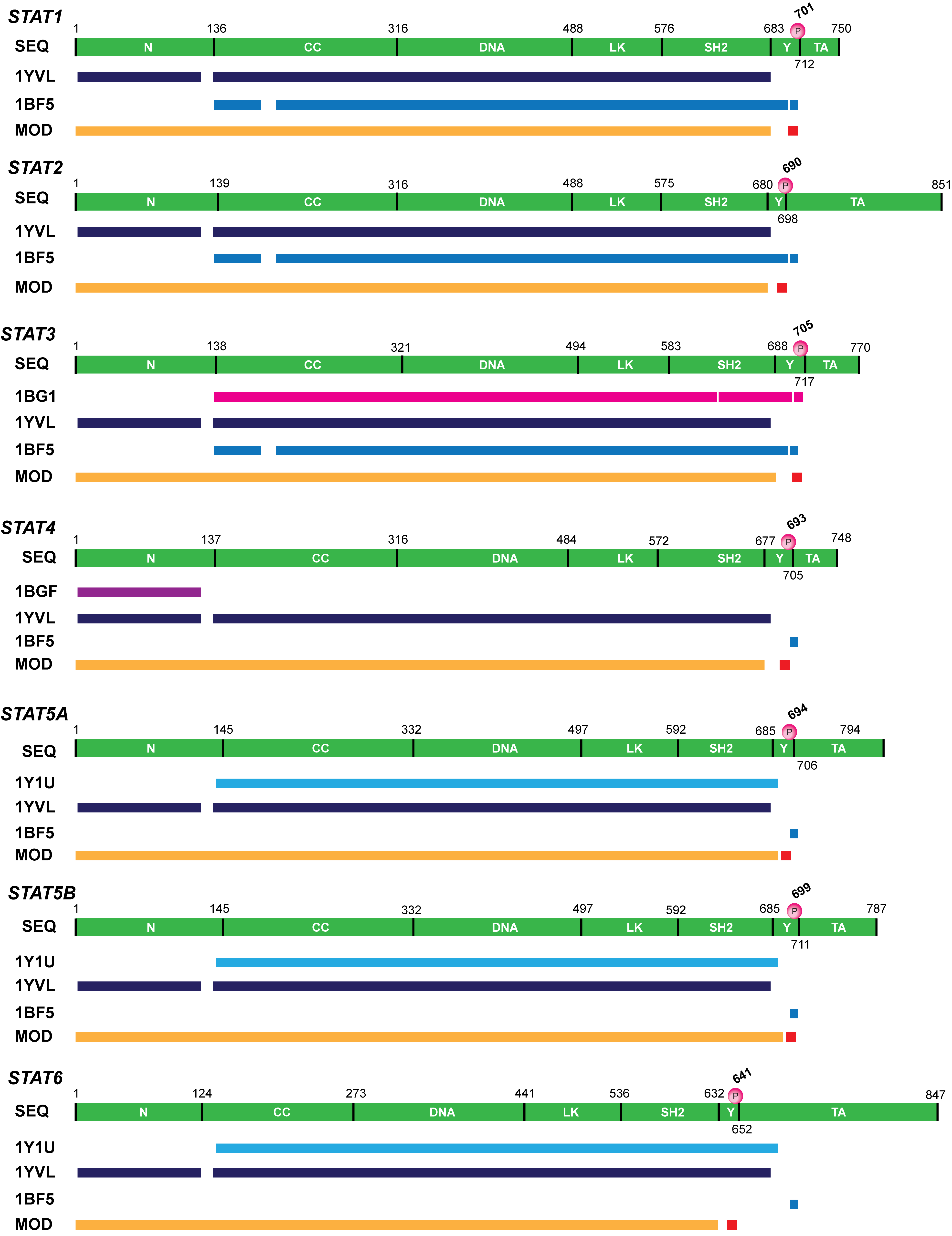

Supplement: S1 Fig — SEQ: full-length sequences with domains, (green)—N: N-terminal domain, CC: Coiled-coil domain, DNA: DNA-binding domain, LK: linker domain, SH2: Src-homology 2 domain, Y: phosphotyrosyl tail segment, TA: transcriptional activation domain; P: phosphorylated tyrosine (pink); MOD: homology models (orange) with phosphopeptides (red); PDB IDs: modeling templates (blue-violet scale). Length of the structures corresponds to the number of amino acids. (TIF) [file pone.0116688.s001.tif]

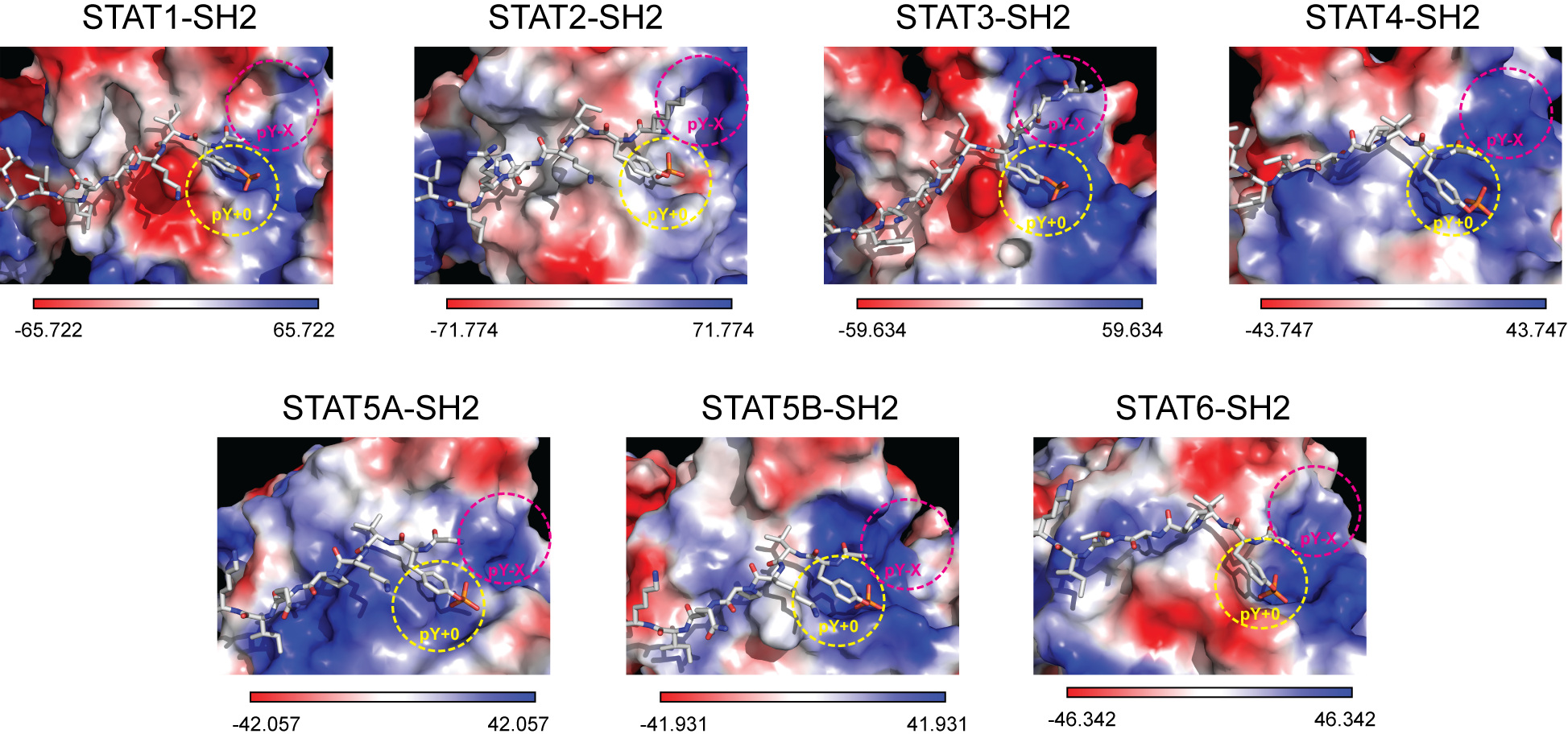

Supplement: S2 Fig — pTyr-peptides are presented in stick representation, pY+0—pTyr binding pocket is encircled by yellow dashed line, pY-X—hydrophobic side-pocket is encircled by pink dashed line. SH2 domains are in the surface representation, colored according to the distribution of the electrostatic surface potential, calculated with APBS. Blue indicates positively charged regions, red indicates negatively charged regions. (TIF) [file pone.0116688.s002.tif]

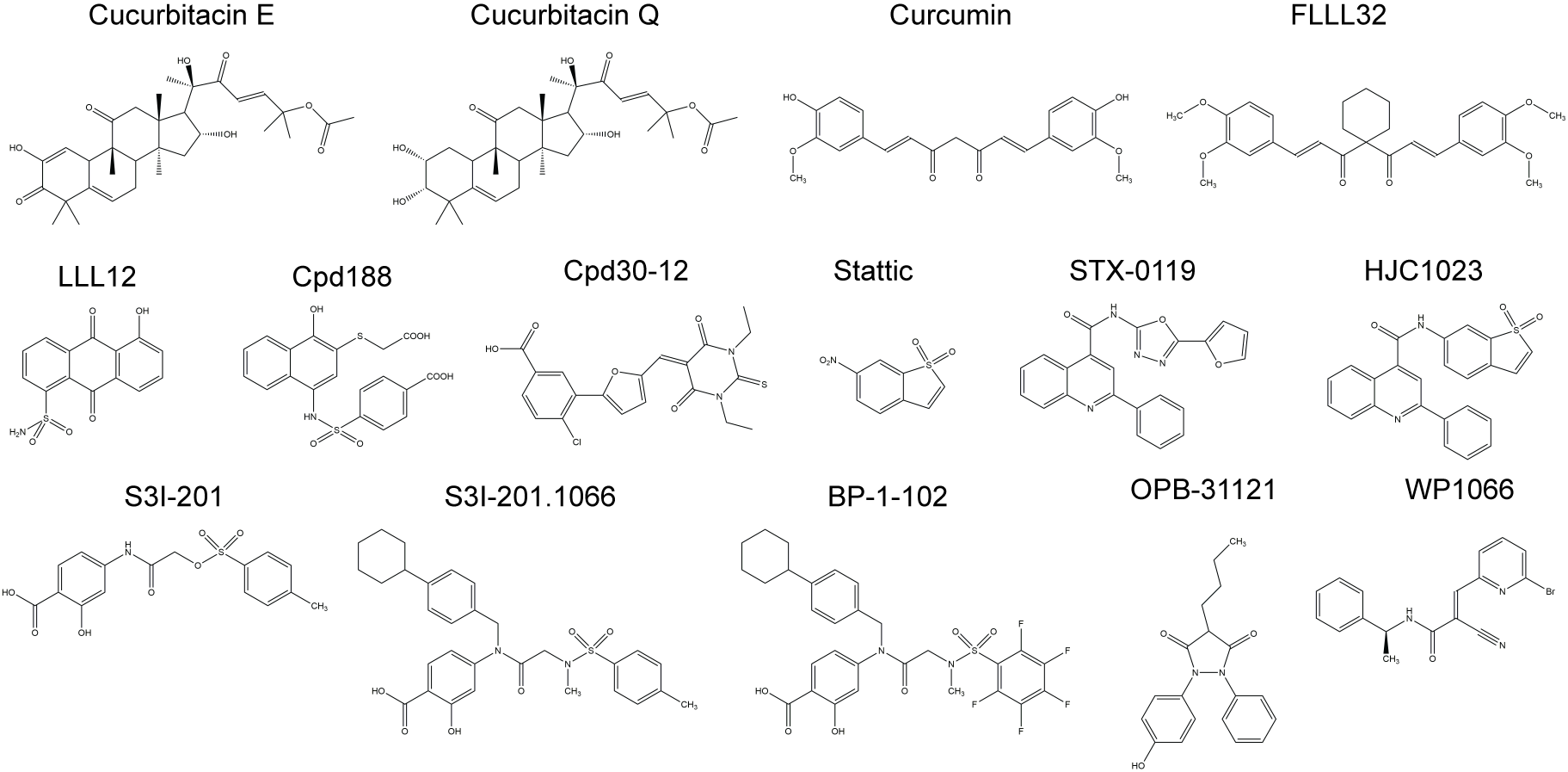

Supplement: S3 Fig — (TIF) [file pone.0116688.s003.tif]

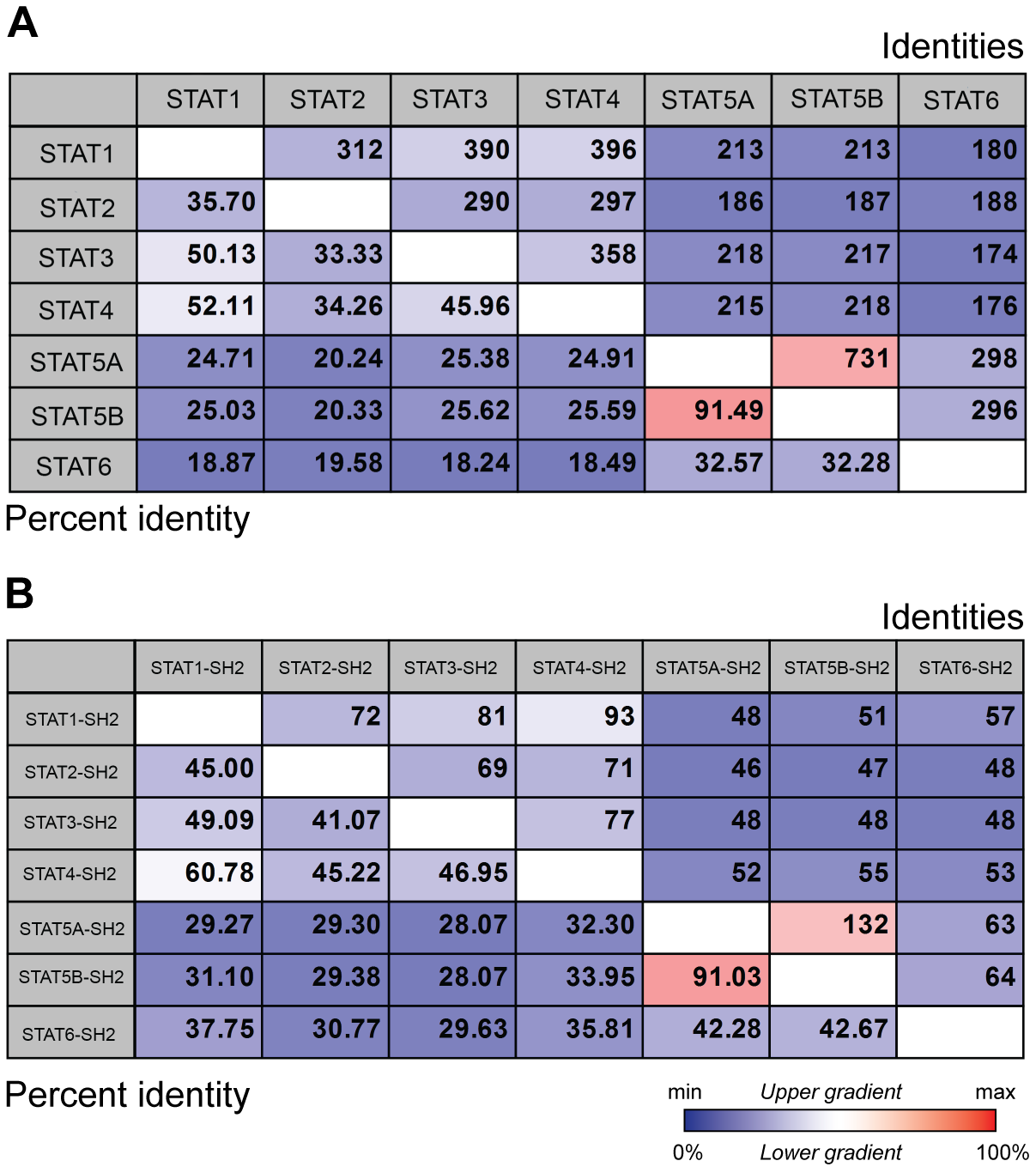

Supplement: S4 Fig — (A) Global comparison of full-length sequences of hSTATs. (B) Local comparison of hSTAT-SH2 domains. Upper comparison presents identities—the number of identical amino acids between two STATs. Lower comparison reflects percent identity of hSTATs. (TIF) [file pone.0116688.s004.tif]

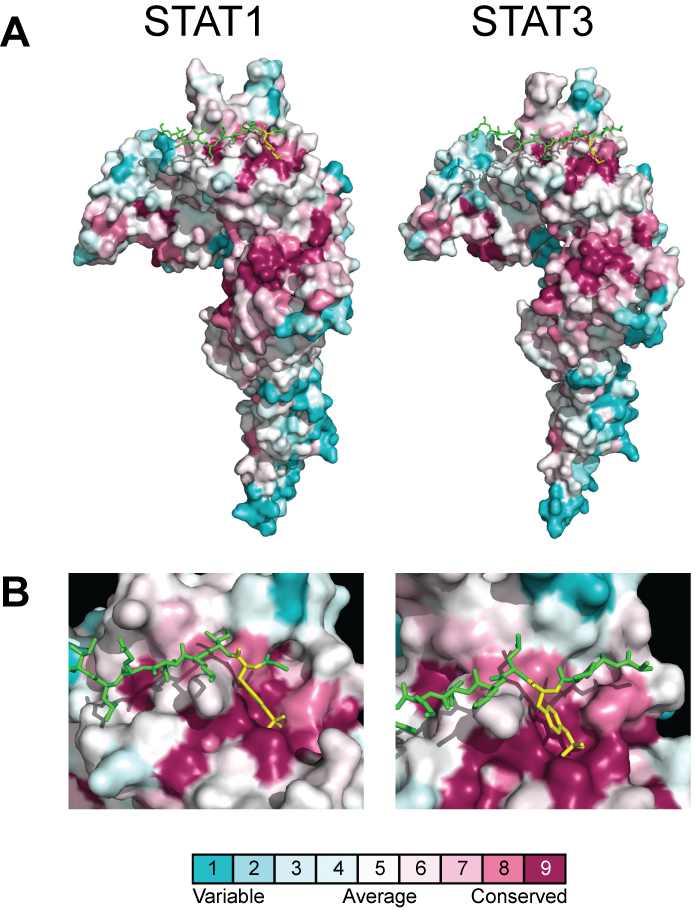

Supplement: S5 Fig — (A) Models of hSTAT1 and hSTAT3 monomers in the surface representation with pTyr-peptides in the stick representation. (B) Models of hSTAT1-SH2 and hSTAT3-SH2 domains in the surface representation with pTyr-linkers, shown as sticks. Structures are colored according to sequence similarity, based on the multiple sequence alignments and phylogenetic relations using ConSurf. Purple indicates conserved residues, white to blue indicate variable residues. pTyr-peptides are colored in green, while pTyr residue is colored in yellow. (TIF) [file pone.0116688.s005.tif]

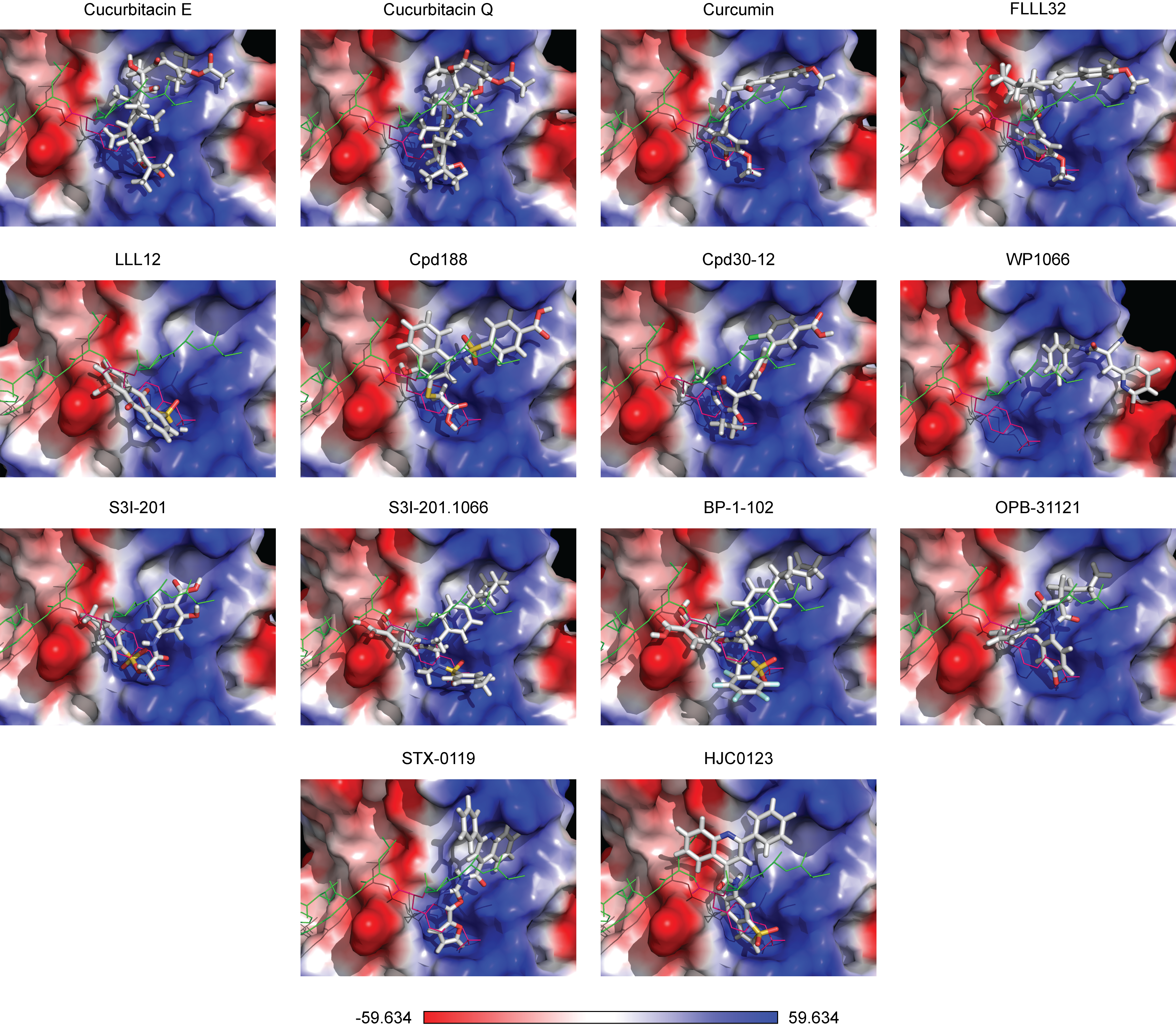

Supplement: S6 Fig — Inhibitors are shown in stick representation, pTyr-linker is presented as lines colored in green with pTyr residue colored in pink. Results were obtained using Surflex-Dock 2.6 program. (TIF) [file pone.0116688.s006.tif]
