# Supplementary material for: Identification of STAT1 and STAT3 Specific Inhibitors Using Comparative Virtual Screening and Docking Validation
Source: PLoS One. 2015 Feb 24;10(2):e0116688. doi: 10.1371/journal.pone.0116688 (PMC4339377; doi:10.1371/journal.pone.0116688)
Supplement: S1 Table — Values were calculated by selected methods of protein homology detection, which are implemented at GeneSilico Metaserver; n/a—no result. (DOCX) [file pone.0116688.s007.docx]

| **Method** | **hSTAT1** | | **hSTAT2** | | **hSTAT3** | | | **hSTAT4** | | | **hSTAT5A** | | **hSTAT5B** | | **hSTAT6** | |
| --- | --- | --- | --- | --- | --- | --- | --- | --- | --- | --- | --- | --- | --- | --- | --- | --- |
|  | 1YVL | 1BF5 | 1YVL | 1BF5 | 1BG1 | 1YVL | 1BF5 | 1BGF | 1YVL | 1BF5 | 1YVL | 1Y1U | 1YVL | 1Y1U | 1YVL | 1Y1U |
| *HHsearch* | 100 | 100 | 100 | 100 | 100 | 100 | 100 | 100 | 100 | 100 | 100 | 100 | 100 | 100 | 100 | 100 |
| *mGenTHREADER* | 4e-35 | 3e-31 | 4e-35 | 4e-25 | 5e-31 | 5e-36 | 1e-24 | n/a | 1e-35 | 1e-29 | 4e-24 | 4e-31 | 7e-24 | 7e-32 | 1e-24 | 2e-24 |
| *COMPASS* | 0 | n/a | 0 | n/a | 0 | 0 | n/a | n/a | 0 | n/a | 0 | 0 | 0 | 0 | 0 | 0 |
