# Supplementary material for: Identification of STAT1 and STAT3 Specific Inhibitors Using Comparative Virtual Screening and Docking Validation
Source: PLoS One. 2015 Feb 24;10(2):e0116688. doi: 10.1371/journal.pone.0116688 (PMC4339377; doi:10.1371/journal.pone.0116688)
Supplement: S2 Table — Based on the multiple sequence alignment and hSTAT models superimposition. Bold—group substitutions of the amino acids in comparison to hSTAT1. (DOCX) [file pone.0116688.s008.docx]

| **SH2 domain** | **Residues involved in the protein-inhibitor interactions** | |
| --- | --- | --- |
|  | pY+0 | pY-X |
| *STAT1* | Lys584, Arg602, Ser604, Glu605, Ser606 | Met579, Gly580, Phe581, Ile582, Ser583 |
| *STAT2* | **Arg583**, Arg601, Ser603, Glu604, Ser605 | Met578, Gly579, Phe580, **Val581**, Ser582 |
| *STAT3* | Lys591, Arg609, Ser611, Glu612, Ser613 | Met586, Gly587, Phe588, Ile589, Ser590 |
| *STAT4* | Lys580, Arg598, Ser600, Glu601, Ser602 | Met575, Gly576, Phe577, **Val578**, Ser579 |
| *STAT5A* | Lys600, Arg618, Ser620, **Asp621**, Ser622 | **Leu595**, Gly596, Phe597, **Val598**, **Asn599** |
| *STAT5B* | Lys600, Arg618, Ser620, **Asp621**, Ser622 | **Leu595**, Gly596, Phe597, **Val598**, **Asn599** |
| *STAT6* | Lys544, Arg562, Ser564, **Asp565**, Ser566 | **Ile539**, Gly540, Phe541, Ile542, Ser543 |
