# Supplementary material for: Identification of STAT1 and STAT3 Specific Inhibitors Using Comparative Virtual Screening and Docking Validation
Source: PLoS One. 2015 Feb 24;10(2):e0116688. doi: 10.1371/journal.pone.0116688 (PMC4339377; doi:10.1371/journal.pone.0116688)
Supplement: S3 Table — Results were obtained using Surflex-Dock 2.6 program. (DOCX) [file pone.0116688.s009.docx]

| **Compound** | **Binding affinity of top-scored conformer** | | | | | | |
| --- | --- | --- | --- | --- | --- | --- | --- |
|  | STAT1 | STAT2 | STAT3 | STAT4 | STAT5A | STAT5B | STAT6 |
| *Cucurbitacin E* | 6.89 | 7.03 | 7.49 | 5.72 | 5.4 | 6.99 | 6.63 |
| *Cucurbitacin Q* | 7.16 | 6.98 | 9.08 | 5.09 | 5.63 | 7.42 | 8.42 |
| *Curcumin* | 7.75 | 7.37 | 7.89 | 7.31 | 4.5 | 7.63 | 7.39 |
| *FLLL32* | 6.72 | 6.67 | 6.74 | 6.71 | 5.73 | 8.51 | 6.75 |
| *LLL12* | 4.79 | 5.35 | 3.95 | 3.8 | 3.34 | 4.32 | 4.97 |
| *Cpd188* | 6.1 | 6.38 | 6.13 | 4.91 | 5.17 | 5.9 | 6.01 |
| *Cpd30-12* | 5.52 | 4.6 | 4.44 | 4.82 | 3.43 | 5.01 | 4.53 |
| *Stattic* | 3.83 | 4.65 | 3.79 | 2.9 | 3.53 | 3.64 | 3.94 |
| *STX-0119* | 4.47 | 4.49 | 4.93 | 5.06 | 4.21 | 4.39 | 5.16 |
| *HJC0123* | 5.71 | 5.45 | 5.27 | 5.13 | 4.15 | 5.99 | 5.79 |
| *S3I-201* | 6.63 | 5.23 | 5.67 | 5.21 | 5.49 | 5.56 | 5.62 |
| *S3I-201.1066* | 8.64 | 6.89 | 7.21 | 7.55 | 5.61 | 9.71 | 7.1 |
| *BP-1-102* | 6.45 | 6.63 | 7.21 | 7.18 | 6.37 | 9.15 | 6.23 |
| *OPB-31121* | 4.11 | 5.22 | 5.64 | 5.13 | 4.77 | 5.41 | 6.18 |
| *WP1066* | 5.37 | 5.09 | 4.81 | 3.5 | 3.98 | 3.72 | 4.65 |
